# ATP and glutamate coordinate contractions in the freshwater sponge *Ephydatia muelleri*

**DOI:** 10.1242/jeb.248010

**Published:** 2025-02-12

**Authors:** Vanessa R. Ho, Greg G. Goss, Sally P. Leys

**Affiliations:** Department of Biological Sciences, University of Alberta, Edmonton, AB, Canada, T6G 2R3

**Keywords:** Porifera, Nervous system evolution, P2X receptor, ATP signalling, Purinergic signalling

## Abstract

Sponges (phylum *Porifera*) are an early diverging animal lineage without nervous and muscular systems, and yet they are able to produce coordinated whole-body contractions in response to disturbances. Little is known about the underlying signalling mechanisms in coordinating such responses. Previous studies demonstrated that sponges respond specifically to chemicals such as l-glutamate and γ-amino-butyric acid (GABA), which trigger and prevent contractions, respectively. Genes for purinergic P2X-like receptors are present in several sponge genomes, leading us to ask whether ATP works with glutamate to coordinate contractions in sponges as it does in other animal nervous systems. Using pharmacological approaches on the freshwater sponge *Ephydatia muelleri*, we show that ATP is involved in coordinating contractions. Bath application of ATP caused a rapid, sustained expansion of the excurrent canals in a dose-dependent manner. Complete contractions occurred when ATP was added in the presence of apyrase, an enzyme that hydrolyses ATP. Application of ADP, the first metabolic product of ATP hydrolysis, triggered complete contractions, whereas AMP, the subsequent metabolite, did not trigger a response. Blocking ATP from binding and activating P2X receptors with pyridoxalphosphate-6-azophenyl-2′,4′-disulfonic acid (PPADS) prevented both glutamate- and ATP-triggered contractions, suggesting that ATP works downstream of glutamate. Bioinformatic analysis revealed two P2X receptor sequences, one of which groups with other vertebrate P2X receptors. Altogether, our results confirm that purinergic signalling by ATP is involved in coordinating contractions in the freshwater sponge.

## Teaser

**Summary:** Nerveless sponges coordinate a sneeze-like reflex using glutamate and ATP signalling to expel water from the body.

## INTRODUCTION

The ability to sense and respond to environmental cues is essential for all forms of life. Most animals use the nervous system for this, but sponges (Porifera) and many multicellular non-animal organisms, e.g. the Venus flytrap (*Dionaea muscipula*) and the aggregating slime mould *Dictyostelium discoideum* (Amoebozoa), carry out sensation and coordination without it. The means by which signals are transmitted between cells to coordinate these activities is as diverse as the organisms themselves, with some quite well known, such as the chloride potential of *Dionaea* (Hodick and Sievers, 1988; Hedrich and Kreuzer, 2023) and cAMP signalling in *Dictyostelium* (Swanson and Lansing Taylor, 1982; Miura and Siegert, 2000), but others still unknown, such as signalling mechanisms for coordinating behaviour in sponges.

Sponges are one of the earliest diverging metazoan lineages (Schultz et al., 2023). Typical animal features such as a mouth and gut and conventionally defined organ systems (e.g. nervous and muscular systems) are absent in sponges. Despite the absence of a nervous system, it is known that all sponges sense and respond to external stimuli (Parker, 1910; de Ceccatty, 1974; Nickel, 2004; Elliott and Leys, 2007). As sessile filter feeders, sponges are susceptible to clogging of their aquiferous canal system with unwanted debris that enters alongside food particles. To address this problem, sponges can control their feeding current; how this occurs depends on the physiological characteristics of the sponge itself. The syncytial tissues of glass sponges (Hexactinellida) (Mackie et al., 1983) propagate electrical currents to coordinate the rapid arrest of the feeding current in response to disturbances (Leys and Mackie, 1997; Leys et al., 1999; Leys, 2003; Tompkins-MacDonald and Leys, 2008). In contrast, multicellular sponges (Demospongiae, Calcarea and Homoscleromorpha) respond by shutting the incurrent pores (ostia) and constricting the aquiferous canal system to slow or halt the feeding current (Leys and Meech, 2006).

Whole-body contractions are a type of behaviour displayed by sponges that function like a sneeze to expel waste and other irritants from the internal canal system (Elliott and Leys, 2007; Kornder et al., 2022). How contractions are coordinated is not well understood: they are too slow to be propagated by electrical impulses, and gap junctions have not yet been identified (Leys, 2015). It was once thought that signal propagation occurred by physically connected cells pulling on each other (Parker, 1910; Pavans de Ceccatty et al., 1960), but this is unlikely because sponges continue contracting despite having torn their tissues (Ludeman et al., 2014). At present, physiological and molecular evidence suggests coordination is accomplished by a paracrine signalling system that may have been a precursor to the nervous system (Fields et al., 2020; Kenny et al., 2020; Musser et al., 2021).

Sponges respond to substances such as l-glutamate and γ-amino-butyric acid (GABA) (Ellwanger et al., 2007; Elliott and Leys, 2010), among others (reviewed in Leys, 2015). Glutamate and GABA, which have diverse signalling and metabolic roles in non-neuronal organisms (Shmukler and Nikishin, 2022), are highly abundant and evolutionarily important neurotransmitters in animal nervous systems (Moroz et al., 2021a; Andersen and Schousboe, 2023). In the freshwater sponge *Ephydatia muelleri*, the former triggers contractions while the latter prevents them (Elliott and Leys, 2010).

In many animal nervous systems, both vertebrate and invertebrate, glutamate signalling often works in concert with adenosine triphosphate (ATP) signalling (Gu and MacDermott, 1997; Hamilton et al., 2008; Shen et al., 2017; Moroz et al., 2021b; Rimbert et al., 2023). As the main energy currency of cells, ATP is both abundant (2–8 mmol l^−1^ in mammalian cells; Traut, 1994) and highly regulated (Dunn and Grider, 2023). These characteristics make it a readily available multi-purpose molecule for fuelling cellular activities and acting as an intercellular chemical messenger (Burnstock, 2009; Rimbert et al., 2023).

Purinergic (ATP) signalling is essential for the function of various tissues, and is a prominent form of communication between glia and neurons (Di Virgilio et al., 2023). ATP in excess of normal cytosolic concentrations is stored in secretory vesicles with other chemical messengers (Burnstock, 2009; Bonora et al., 2012). In the nervous system, it is co-released with other neurotransmitters (Fellin et al., 2006; Burnstock and Verkhratsky, 2009) in response to membrane stretching, glutamate transmission or even by ATP signalling itself (Fields and Burnstock, 2006). Activation of the P2X receptor channels by extracellular ATP allows cations, namely Na^+^, K^+^ and Ca^2+^, to pass into the cell (North, 2016). This in turn leads to a complex cascade of downstream effects including, but not limited to, Ca^2+^ signalling (Hamilton et al., 2008), glutamate release (Nakatsuka et al., 2003) and subsequent autocrine and paracrine functions (Hinoi et al., 2004), and more ATP release (Shen et al., 2017).

The nervous system-like characteristics of coordination in multicellular sponges led us to ask whether purinergic signalling by ATP plays a role in coordinating the behaviour of sponges. We took advantage of the ease of laboratory culture and transparent tissues of the freshwater species *Ephydatia muelleri* [Demospongiae (Lieberkühn 1856)]. Using a pharmacological approach, we tested the hypothesis that sponges respond to exogenous ATP in a concentration-dependent manner, and that ATP signalling is triggered after glutamate signalling. We also carried out a phylogenetic study of sponge P2X receptors, examining the evolutionary relationships of P2X receptors in metazoans and non-metazoans. The evidence we provide from both pharmacological and bioinformatic approaches bridges a gap in knowledge in the physiology of sponges, addressing the question of how cells communicate across the body of the sponge to coordinate behaviour.

## MATERIALS AND METHODS

### Sponge collection and culture

**Fig. 1.**
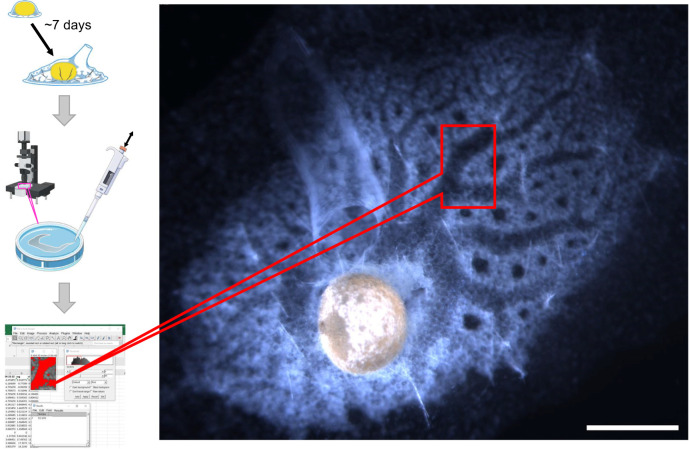
**Overview of the experimental workflow and top view of a representative stage 5 juvenile sponge, *Ephydatia muelleri*.** After hatching and culturing sponges, time-lapse images of contraction responses to mechanical (not shown) or chemical stimulation were taken on a stereomicroscope. Image analysis was used to measure a selected segment of the excurrent canal system and processed to determine the average relative change in canal area. Scale bar: 0.5 mm.

Fragments of *E. muelleri* containing gemmules were collected in the winter months of 2020 and 2021 from O'Connor Lake, BC, Canada. These were stored in unfiltered lake water in the dark at 4°C in plastic bags, aerated monthly, until use. Cleaning and culturing were done as described previously (Leys et al., 2019). Briefly, gemmules were mechanically dissociated from the dead sponge skeleton in ice-cold water and sterilized with 1% hydrogen peroxide. Sponges were cultured on flame-sterilized 22 mm^2^ glass coverslips in ∼20 ml Strekal's medium pH 7.4 (Strekal and McDiffett, 1974) in 60 mm diameter Petri dishes and kept in the dark; 50% water changes were done every 2–3 days until 6–10 days post-hatching (stage 5). Sponges with a fully formed and functional aquiferous canal system and a single, upright osculum (Fig. 1) were used for experiments.

### Test substance preparation and application

Stock solutions of pharmacological substances were prepared with Milli-Q water (Millipore-Sigma) in the following concentrations: 100 mmol l^−1^ adenosine 5′-triphosphate (ATP) disodium salt hydrate (Sigma-Aldrich A2383), 20 mmol l^−1^ l-glutamate (Sigma-Aldrich G1251), 100 mmol l^−1^ PPADS (Sigma-Aldrich P178) and 200 units mg^−1^ apyrase (Sigma-Aldrich A6410). ADP and AMP, prepared at 100 mmol l^−1^ each, were obtained from Sigma-Aldrich (A2754 and A1752). Using pH indicator paper, we tested whether the addition of drugs to the solution would change the pH and cause sponges to sneeze, and found no changes.

Prior to filming, the culture medium was removed until 10 ml was left in the Petri dish. The sponges were then carefully set on the microscope stage to prevent the triggering of a premature contraction. All sponges were filmed for 5 min prior to the application of any substance to ensure no contraction was occurring. If sponges were already in the middle of a contraction, they were left alone for at least 1 h to allow the completion of the contraction before proceeding with the experiment.

Dilution of test chemicals was calculated to a final volume of 10 ml in the bath application, and was carried out using the previously withdrawn medium from the Petri dish to make 1 ml of 10× solution. This was then carefully mixed around the dish by gently pipetting 7–8 times, ensuring sponges were fully and evenly exposed to the test substance. To validate the mixing technique, fluorescein dye (134 mg l^−1^, 0.2 µm filtered twice) was used as a visual equivalent (not shown). For a negative control, 1 ml of the withdrawn Strekal's medium was mixed back into the Petri dish. Sponges treated with multiple substances (apyrase and PPADS experiments) were first bathed in the indicated drug and left undisturbed for 20 min prior to the application of glutamate or ATP.

### Digital time lapse and data acquisition

Sponges were viewed with a stereomicroscope (Olympus SZX-12) and digital time-lapse images were captured with a QI-Cam colour CCD camera (Retiga QImaging) and Northern Eclipse version 7 (Empix Imaging, Toronto, ON, Canada). Images were saved as 8-bit greyscale except for PPADS experiments, which were captured in 16-bit colour for splitting colour channels. Single-treatment time-lapse images were captured at 20 s intervals for 60 min; dual-treatment time-lapse images were captured at 30 s intervals for 90 min. Behavioural responses were analysed and quantified using FIJI ImageJ version 1.53t (Schindelin et al., 2012) by measuring changes in the excurrent canal area during a contraction (Ho and Leys, 2024). Canal sections undergoing the most visually distinguishable changes in size during the course of a contraction were selected and measurements were taken as the percentage change in area. The data were exported to MS Excel (2016) to calculate the mean relative change in area after application of the stimulus. Statistical differences between responses of sponges in different treatment groups (maximum absolute percentage change in canal area) were calculated with one-way ANOVA with *post hoc* Tukey–Kramer HSD test using GraphPad Prism (v.10.2.3 for Windows; see Dataset 1). Graphs were generated in MS Excel (2016) or GraphPad Prism (v.10.2.3).

### Bioinformatic analysis and phylogenetic tree building

The protein sequences for P2X-like receptors in *E. muelleri* were obtained by identifying the most highly expressed genes from RNAseq differential transcript expression data (Kenny et al., 2020; available from EphyBase: https://spaces.facsci.ualberta.ca/ephybase/). The differential transcript expression values of P2X receptors in *E. muelleri* showed low relative transcript abundance. We selected the two most highly expressed genes of the group: *Em0004g77a* and *Em0004g666a* (FPKM 7.37 and 3.15, respectively; Table S1). The corresponding protein sequences were obtained from functional annotation results (Kenny et al., 2020; also available from EphyBase) and verified by performing a BlastP search (NCBI).

P2X receptor sequences from diverse metazoan and non-metazoan lineages were collected by searching the NCBI protein database (Table S2). Additional sponge P2X-like receptors (*Spongilla lacustris*, *Aphrocallistes vastus*, *Sycon coactum* and *Eunapius fragilis*) were identified by tBLASTn against their respective transcriptomes (Riesgo et al., 2014; Windsor-Reid et al., 2018; data available from https://leyslab.weebly.com/data-available.html). Protein sequences <200 amino acids were excluded (Fig. S1).

Sequences were aligned with MAFFT v.7 (Katoh et al., 2017) and trimmed with TrimAl v.1.3 (Sánchez et al., 2011). SeaView v.5 (Gouy et al., 2021) was used to visualize the aligned sequences and fragmented or poorly aligned sequences were removed manually. The phylogenetic tree was computed on IQ-Tree using 1000 ultrafast bootstrap approximations (Hoang et al., 2017), and the resulting consensus tree was viewed with Fig Tree v.1.4.4 (http://tree.bio.ed.ac.uk/software/figtree/) and rooted with the *D. discoideum* P2X sequence.

The predicted 3D protein structures of selected *E. muelleri* P2X receptor subunits, Em0004g77a and Em0004g666a, were compared with the well-characterized structure of the *Danio rerio* P2X4 subunit (NCBI NP_945338.2), all of which were generated on Phyre2 (Kelley et al., 2015) and visualized on EzMol (Reynolds et al., 2018). Consensus sequence shading of phylogenetic tree P2X receptor sequences was made with Boxshade (https://junli.netlify.app/apps/boxshade/) at 0.75 sequence agreement (range 0–1) (Fig. S2).

## RESULTS

### Variations in contraction responses to mechanical or chemical stimulation

**Fig. 2.**
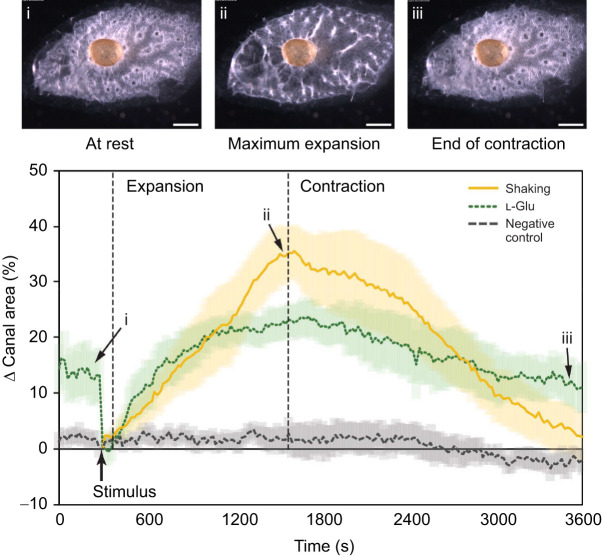
**Progression of one contraction cycle in response to mechanical or chemical stimulation.** Images show the stages a contraction (i–iii) in sponges over the course of a contraction (scale bar: 0.5 mm). Corresponding labels and arrows on the graph indicate the time and canal size of the contraction stage shown by the images. Changes in excurrent canals in response to stimulation are plotted as follows: physical shaking (solid yellow line), 70 µmol l^−1^ l-glutamate (dotted green line) or negative control (dashed grey line); shading behind plotted lines indicates the standard error of the mean. *n*=7 individuals for each treatment.

Sneezes triggered by endogenous signals (i.e. without application of chemicals) were induced by shaking Petri dishes vigorously, which provided a reference for how excurrent canals change in size, first expanding and subsequently contracting (Fig. 2). We follow previous researchers in referring to this as a ‘sneeze’ (Elliott and Leys, 2007; Ludeman et al., 2014; Colgren and Nichols, 2022) and in our work we recorded the dilation of the excurrent canals as the expansion phase of the sneeze, and the constriction of the excurrent canals as the contraction phase of the sneeze. It is generally considered that the opposite reaction is occurring in the incurrent canals during this process. To trigger a sneeze pharmacologically, we added 70 µmol l^−1^ l-glutamate (Movie 1). In shaken sponges, expansion of excurrent canals started at one end of the sponge near the osculum, and moved in a wave-like manner across the body of the sponge, whereas pharmacological stimulation caused all canals to respond simultaneously. In both cases, the percentage change in area showed excurrent canals expanding and constricting, together making one ‘contraction cycle’ (Fig. 2). Mixing the medium with pipetting did not trigger sponge contractions.

### ATP treatment

**Fig. 3.**
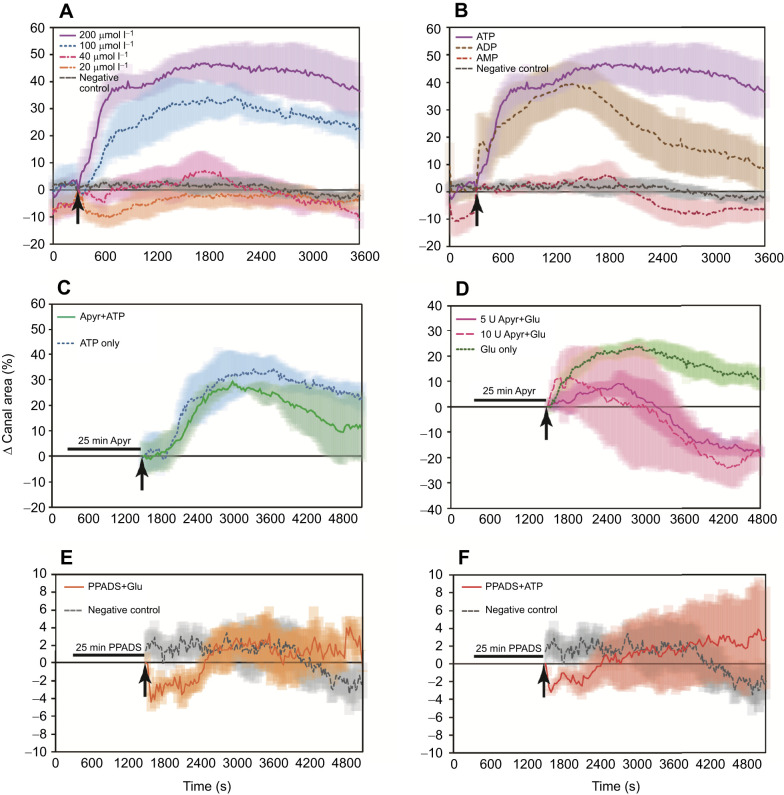
**Pharmacological treatments have different effects on the contraction response.** (A) Responses to ATP are concentration dependent. While 20 and 40 µmol l^−1^ ATP cause a temporary constriction of excurrent canals, 100 and 200 µmol l^−1^ ATP trigger the rapid expansion of excurrent canals but prevent the constriction phase to complete a contraction cycle. (B) Sponges respond to 100 µmol l^−1^ ATP and ADP, but not AMP. (C) Addition of 5 U ml^−1^ apyrase (Apyr) to the dish medium allows for a complete contraction when triggered by 100 µmol l^−1^ ATP. (D) Addition of 5 or 10 U ml^−1^ apyrase causes the excurrent canals to become constricted when 70 µmol l^−1^ l-glutamate is added. (E,F) PPADS blocks either 70 μmol l^−1^ glutamate- (E) or 100 μmol l^−1^ ATP-induced contractions (F) from occurring. Shading behind plotted lines represents the standard error of the mean. *n*=7 individuals for each treatment in A, *n*=5 in B–F (*n*=4 for 10 U ml^−1^ apyrase+glutamate in D).

Sponges responded to ATP in a dose-dependent manner. Application of lower doses of ATP (20 or 40 µmol l^−1^) caused a small constriction of the excurrent canals (Fig. 3A). At the higher concentration of 40 µmol l^−1^ ATP, excurrent canals eventually expanded slightly (<10%) before relaxing to the initial resting size, whereas 20 µmol l^−1^ ATP caused a slight constriction of excurrent canals and slow relaxation, but no expansion. High concentrations of exogenous ATP (100 and 200 µmol l^−1^) caused the rapid and intense dilation of excurrent canals and prevented their constriction. These dosages triggered responses so strong that some sponges tore the ‘tent’ which forms the outer tissue of the sponge. At 200 µmol l^−1^ ATP, expansion of the excurrent canals was faster and more intense than with 100 µmol l^−1^ ATP (Fig. 3A), with more sponges tearing their surface tissues than for those exposed to lower concentrations. The most severe responses caused the aquiferous canals themselves to tear (Movie 2).

### Pre-treatment with the hydrolytic enzyme apyrase

Whereas treatment with 100 µmol l^−1^ ATP caused the excurrent canals to remain expanded, pretreatment with 5 U ml^−1^ apyrase allowed 100 µmol l^−1^ ATP-treated sponges to complete a full expansion and contraction cycle (Fig. 3C). Treatment with 70 µmol l^−1^ glutamate after pre-incubation in apyrase caused sponges to rapidly expand and contract their excurrent canals, and by the end of the experiment, the excurrent canals were more constricted than at the beginning of the experiment (Fig. 3D). Increasing the apyrase concentration to 10 U ml^−1^ caused a greater variability in the responses, and some sponges sneezed in response to the apyrase itself. At the end of the experiment, the excurrent canals of sponges treated with 10 U ml^−1^ apyrase were more constricted than those of sponges pre-treated with 5 U ml^−1^ apyrase (Fig. 3D). Sneeze responses (expansion and constriction of the excurrent canals) in sponges pre-treated with apyrase were more intense than those in sponges treated with glutamate only, and the contractions travelled in a wave-like manner from one end of the sponge body to the other. However, when ATP was added in the presence of apyrase, the tissues did not tear apart as they did when only treated with ATP.

Furthermore, apyrase itself appeared to have an effect on the excurrent canals. In all experiments, sponges were observed to respond when apyrase was added to the Petri dish. Sponges briefly expanded and contracted their excurrent canals, while some did the opposite, constricting then expanding the canals. This occasionally triggered a sneeze that propagated across the body of the sponge in a wave-like manner similar to sneezes triggered by mechanical stimulation.

### Treatments with ADP and AMP

Sponges responded rapidly to treatment with ADP, and the amplitude of the expansion phase (excurrent canal dilation) was nearly identical to responses triggered by the equivalent concentration of ATP (*P*>0.05; Fig. 3B, Dataset 1). However, a significant difference was that ADP triggered a complete contraction cycle, while the same concentration of ATP immobilized the excurrent canals in an expanded (dilated) state. Contractions triggered by either ATP or ADP were vigorous, but none of the sponges stimulated by ADP tore their tissues. Sponges did not respond at all to AMP (Fig. 3B).

### Pre-treatment with PPADS

Sponges treated with 100 µmol l^−1^ PPADS could not be stimulated to sneeze with 70 µmol l^−1^ glutamate (Fig. 3E) or 100 µmol l^−1^ ATP (Fig. 3F; Movie 3). The activity of the excurrent canals in both of these treatment groups was significantly reduced compared with that in the same experiment without PPADS (*P*<0.0001 for glutamate with or without PPADS and *P*<0.005 for ATP with or without PPADS; Dataset 1). The absolute maximum percentage change in excurrent canal area of treatments with PPADS was not statistically or visually different from that of the negative controls (*P*<0.0001 and *P*<0.005 for glutamate+PPADS and glutamate+ATP, respectively), with only subtle and gentle movements of parts of the sponge.

### Bioinformatic analysis of purinergic receptors

**Fig. 4.**
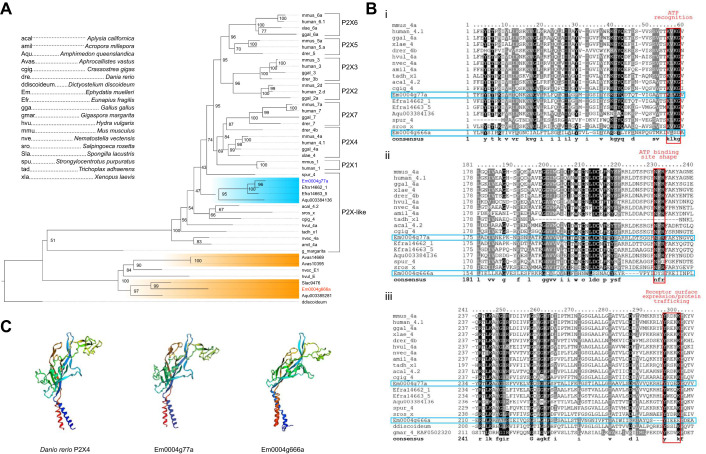
**Bioinformatic analysis of *E. muelleri* P2X receptor sequences.** (A) Consensus tree of evolutionary relationships of metazoan and non-metazoan P2X and P2X-like receptor amino acid sequences, rooted to the aggregating slime mould *Dictyostelium discoideum*. Phylogeny was generated in IQ-TREE v.1.6.12; branch support values were calculated using 1000 ultrafast bootstrap replicates shown as numbers (0–100) in nodes. Sponge sequences are highlighted and *E. muelleri* sequences are labelled in blue (Em0004g77a) and red (Em0004g666a). Branch lengths are not representative of time scales. (B) Boxshade multiple sequence alignment of one P2X receptor sequence from each organism; sequence agreement fraction was set to 0.75 and consensus sequence text is bolded. Only sequences with 100% bootstrap support values in relation to vertebrate P2X sequences are shown (except for iii). The complete set of shaded sequences is available in Fig. S2. P2X conserved motifs are outlined in red and are listed from N to C along the protein sequence: (i) KxKG, (ii) NFR, (iii) YxxxK. (C) 3D models of P2X receptor subunits of *Danio rerio* (RefSeq NP_945338.2) and *E. muelleri* show highly similar predicted protein structures, although Em0004g666a was not found to have the characteristic P2X conserved motifs in its amino acid sequence.

P2X receptors were found in all branches of Amorphea (Adl et al., 2012) in the eukaryotic domain of life (Fig. 4A). Rooted to *D. discoideum* P2X, our tree showed two distinct branches of P2X receptors: one with only invertebrates and another that included both vertebrate and invertebrate P2X receptors (Fig. 4B). The group containing both vertebrate and invertebrate P2X-like receptors shared strong sequence consensus both in the characteristic motifs of P2X receptors (North, 2002) and in sharing many amino acid sequences along the length of the protein (Fig. 4B; Fig. S2).

Predicted 3D constructions of the receptor subunit showed structural similarities between both *E. muelleri* sequences and the crystal structure of a P2X receptor from a zebrafish (Kawate et al., 2009), as well as a 3D model of a P2X receptor from the sea slug *Aplysia californica* (Györi et al., 2021), all sharing the so-called ‘dolphin’-shaped structure that is characteristic of vertebrate P2X receptors (Fig. 4C). Em0004g77a shared conserved amino acid sequences with well-studied vertebrate and invertebrate P2X receptors, and many of these protein sequences also shared many amino acid sequences outside of these conserved motifs. In contrast, Em0004g666a lacked the consensus sequences for ATP binding and recognition sites, and only had a partial match to the conserved protein trafficking sequence KxKG (Fig. 4Biii). However, the trafficking sequence was present in amoeba (*D. discoideum*) and fungus (*Gigaspora margarita*) P2X sequences (Fig. 4Biii).

## DISCUSSION

The signalling pathways that coordinate behaviour in sponges parallel many of the specialized physiological systems of vertebrates and are highly conserved in the evolutionary history of animals. Our data show that ATP signalling is involved in coordinating the contraction behaviour in sponges because sponges respond to ATP in a concentration-dependent manner. Moreover, our data indicate that purinergic signalling by ATP is necessary for triggering the expansion phase of the sneeze response and that ATP works together with glutamate signalling to coordinate the contraction phase of the sneeze behaviour. The balance of these chemical signals is important for propagating the contraction across the tissues to expel fluid from the sponge aquiferous system.

### ATP signalling is involved in coordinating contractions

We first investigated whether ATP would induce a response in sponges, and how it might affect the sponge sneeze behaviour. The transcriptome of *E. muelleri* contains several P2X receptors (https://spaces.facsci.ualberta.ca/ephybase/) (Table S1), so we expected to find that ATP would trigger a behavioural response from the sponge. Part of our initial hypothesis was that ATP acts downstream of glutamate. As glutamate can trigger a sneeze (Elliott and Leys, 2010), we thought that glutamate caused the expansion of excurrent canals in the first half of the expansion–contraction cycle, and that ATP was responsible for ending the contraction by constricting excurrent canals. We therefore expected to see the excurrent canals constrict in the presence of exogenous ATP, essentially quenching the sneeze behaviour. However, while sponges do have a specific ATP-induced response, we found that with enough ATP, the excurrent canals expanded and were immobilized in this dilated state instead.

As with most cellular receptors, P2X receptor subtypes each have their own ligand-binding affinities and kinetic properties (North, 2002). Structural variations between P2X receptors caused by differences in amino acid sequence result in different pharmacological and kinetic properties (Chataigneau et al., 2013; Györi et al., 2021). Our data show that sponges responded in a concentration-dependent manner to ATP, suggesting different subsets of P2X receptors were activated and caused different effects. We found that at high doses of ATP (100 and 200 µmol l^−1^), excurrent canals expand vigorously, and at lower concentrations (20 and 40 µmol l^−1^), they appear to constrict. At 40 µmol l^−1^ ATP, the excurrent canals first constrict before gradually expanding and contracting, the latter resembling a slow, smaller sneeze-type behaviour (Fig. 3A). This suggests that there may be a threshold for ATP-induced activation of the activities that trigger the start of a sneeze.

### ATP and glutamate have different roles in coordinating contractions

In the nervous system, there are two ligand-gated receptor families that mediate postsynaptic cellular activities: ionotropic receptors and G-protein-coupled (metabotropic) receptors (Grubbs, 2008). Whereas P2X receptors are ionotropic, allowing direct influx of ions into the cell when activated by ATP, metabotropic glutamate receptors involve several downstream steps to transduce chemical signals (Hamilton et al., 2008). Our experiments showed that hydrolysis of extracellular ATP by apyrase modulated the pharmacologically stimulated sneeze behaviour. While adding ATP alone caused the excurrent canals to expand and remain expanded, when ATP was added to the sponge in the presence of apyrase, the sponge was able to complete the full expansion–contraction cycle (the sneeze).

After ATP is released by cells, the purinergic signal is terminated by degradation of ATP by ectonucleotidases (Burnstock and Verkhratsky, 2009). When adding a high concentration of ATP to sponges, a sneeze is triggered, but it appears as though the receptors are overstimulated and the ATP overwhelms endogenous removal mechanisms, thus causing the sustained expansion of excurrent canals. Therefore, pre-incubation with the ectonucleotidase apyrase aids in the breakdown of ATP and ends the otherwise sustained signal transmission from excess ATP (Del Campo et al., 1977).

Individual sponges reacted differently to apyrase, resulting in varied responses such as premature contractions (data not shown). Apyrase is highly efficient at metabolizing ATP (Del Campo et al., 1977), and may be metabolizing the endogenous ATP that the sponge normally releases to control its pumping activity (Györi et al., 2021), causing an imbalance in the endogenous signalling feedback loops. Stimulation with glutamate causes dilation and then constriction of excurrent canals, but at the end of the sneeze, the excurrent canal diameter was usually smaller than when the sponges were at rest. This effect was more pronounced when sponges were treated with the higher concentration of 10 U ml^−1^ apyrase, and the higher enzymatic content also increased the variability of sponge responses (Fig. 3D). As with all physiological systems, sponges rely on a balance of signalling inputs and outputs to regulate their internal activities and maintain homeostasis. Our results suggest that ATP and glutamate work in the same pathway to coordinate contractions, but play different roles: ATP seems to cause the expansion of excurrent canals, whereas glutamate functions to constrict them.

### Contractions require ATP signalling

We then asked whether ATP is necessary for contractions or whether it has a more modulatory role in contractions. PPADS, a selective P2 receptor blocker (Lambrecht et al., 1992), has been previously shown to block activation of P2X receptors by ATP in other invertebrate organisms (Agboh et al., 2004; Györi et al., 2021). We found that sponges treated with 100 µmol l^−1^ PPADS did not show any response when stimulated with 70 µmol l^−1^ glutamate or 100 µmol l^−1^ ATP. This result strongly suggests that ATP is required to activate purinergic receptors in order to initiate a sneeze; it also shows that ATP signalling is triggered after the glutamate signal. We found that using a lower concentration of PPADS (10 µmol l^−1^ PPADS) with 100 µmol l^−1^ ATP did not block the sneeze response (data not shown).

One question that arises is where P2X receptors might be localized in the sponge. We assumed that as the entire sponge choanosome is constructed of choanocyte chambers uniformly distributed around the terminal branches of incurrent canals that fill every space within that tissue, then P2X receptors would be localized somewhere on the incurrent canals, potentially adjacent to chambers. To determine this, we attempted *in situ* hybridization with the two P2X receptor genes using our published protocols (Ho et al., 2024). While control genes for Silicatein M4 worked well in terms of labelling cells that produce siliceous spicules, we found no fluorescence expression of the two P2X receptor genes (data not shown). As P2X receptor expression is extremely low in our RNAseq data (FPKM 7.37 and 3.15 of the two most highly expressed P2X receptor transcripts; Dataset 1), we expect that it would be very difficult to see fluorescence in the tissues without using a more sensitive method, such as hybridization reaction chemistry (Dirks and Pierce, 2004).

### Proposed pathway for signalling by ATP and glutamate

Activation of P2X receptors triggers and modulates glutamate release, and glutamate signalling also affects ATP signalling (Gu and MacDermott, 1997; Fields and Burnstock, 2006). In neurons, antagonizing P2X receptors prevents enhanced glutamate signalling (Nakatsuka et al., 2003). Glutamate signalling in astrocytes causes the release of ATP, which stimulates propagation of Ca^2+^ signals (Hamilton et al., 2008). In the freshwater pond snail *Lymnaea stagnalis*, neurons increase ATP release when stimulated by neurotransmitters (Gruenhagen et al., 2004), and ATP appears to play a role in modulating glutamate sensitivity in the leech (*Hirudo medicinalis*) CNS as well as increasing intracellular Ca^2+^ (Müller et al., 2000). As shown in our data, it is likely that this complex relationship between ATP and glutamate exists in sponges to coordinate the sneeze behaviour, and that this system was integrated into the evolution of the nervous system.

**Fig. 5.**
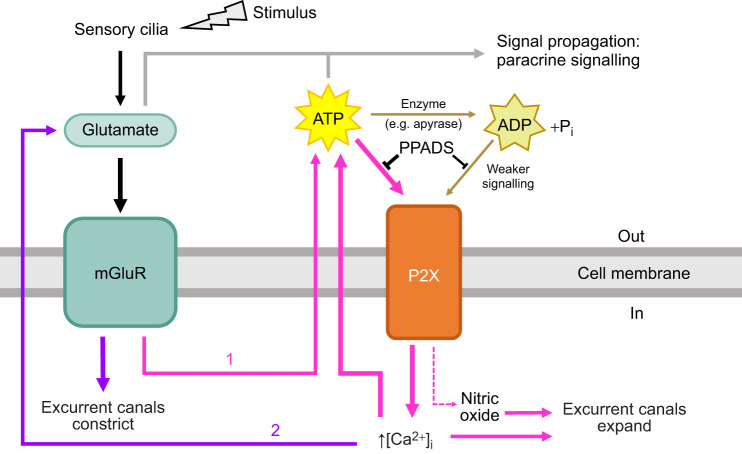
**Proposed signalling pathway demonstrating the roles of l-glutamate and ATP in controlling contractions (sneezes).** A mechanical or chemical stimulus activates sensory cilia, triggering the release of glutamate. Metabotropic glutamate receptors (mGluRs) on contractile cells lining the aquiferous canal system become activated and trigger the release of ATP (1). ATP increases intracellular calcium levels and nitric oxide to expand the excurrent canals. Ca^2+^ also aids in signal amplification and propagation by causing the release of more signalling molecules (e.g. ATP and glutamate) (2). ATP and glutamate may work in both a paracrine and autocrine manner. Eventually, this signal for excurrent canal expansion will weaken as ATP is removed from the extracellular space and the signal for constricting the excurrent canals will take over, allowing the contraction to come to an end.

We propose that a similar pathway regulates contraction behaviours in sponges, as described in Fig. 5. A stimulus, either mechanical or chemical, is detected by sensory primary cilia lining the osculum, the chimney-like structure through which water is expelled from the sponge (Ludeman et al., 2014). These stimuli could result from clogging of the canals with sediment, causing mechanical irritation or reduced flow through the sponge, or by intake of unwanted particles (Kornder et al., 2022). The cilia in the osculum are analogous to the primary cilia of the kidney epithelium in function (Singla and Reiter, 2006): changes in water flow change the bending of the cilia, triggering entry of Ca^2+^ into the cilium, and thereafter the release of signalling molecules such as glutamate from pinacocyte epithelia. Glutamate, acting through metabotropic glutamate receptors, then causes a cascade of downstream effects: the release of ATP, which acts in both an autocrine and paracrine manner to increase intracellular calcium via ionotropic P2X receptors and mobilization of internal calcium stores. This would then activate the contractile apparatus in cells, expanding the excurrent canals by the contraction of incurrent canals. Nitric oxide (NO) activity is likely also activated by ATP signalling in sponges as it is in other animals (Burnstock and Verkhratsky, 2012) and aids in tissue modulation: NO donor NOC-12 triggered the sneeze response in *Tethya wilhelma* (Ellwanger and Nickel, 2006) and *Spongilla lacustris* (Ruperti et al., 2024). Specifically, excurrent canals of *S. lacustris* were shown to expand in the presence of NO. Eventually, through the breakdown or reuptake of ATP, the cue for dilating the excurrent canals diminishes while the metabotropic glutamate-driven signal for constricting the excurrent canals takes over.

Despite blocking sneezes with PPADS, the pumping activity of the flagellated chambers did not stop. Therefore, it is possible that contractions are regulated separately from the sponge's basal pumping activity, although this requires further investigation.

### P2X receptors are evolutionarily conserved

Sponge P2X receptors sequences share high sequence homology with those of well-characterized P2X receptors. In fact, these receptors, though notably absent in insects and the nematode *Caenorhabditis elegans*, are highly conserved among most animal lineages (Fountain and Burnstock, 2009). P2X receptors themselves have been identified in non-metazoan eukaryotes including fungi, amoeba and green algae (Fountain, 2013). It is plausible that P2X receptors had a role in the advent of aggregating/colonial behaviours by mediating intercellular communication, and became integrated into a system of coordination in the first metazoans. As animals evolved increasing morphological complexity (e.g. defined organ systems), the role of P2X receptors also expanded.

In vertebrates, several P2X receptor subtypes have been characterized, each with their own unique properties and roles in various physiological systems (North, 2016). Our results show that invertebrates also have several P2X receptor subtypes of their own (Fig. 4A). Many of these sequences have high amino acid sequence similarity in the conserved motifs and functional domains as vertebrate P2X receptors (Fig. 4B, labelled ‘P2X-like’). Regions of conserved amino acid residues are present in most of the sequences we analysed, but not all of these receptor sequences contain the distinguishing conserved motifs of P2X receptors (e.g. Em0004g666a; Fig. S2). It is likely that these highly conserved residues and regions are essential in retaining basic purinoceptor structure and function, and were present in the ancestral form of P2X receptors. Our phylogeny shows two distinct sets of receptors: one group that includes the vertebrate P2X receptors and another that appears to be exclusive to invertebrates. Whereas the former all contain the conserved amino acid sequence motifs that are characteristic of P2X receptors, these sequence motifs are not present in the latter (Fig. S2). The phylogenetic tree is rooted to the amoebozoan *D. discoideum*. Though the predicted phylogeny shows *D. discoideum* has no relationship to other P2X receptors, our multiple sequence alignment shows that 1 of 3 conserved motifs is present, along with other highly conserved domains. The presence of one of the conserved motifs in the P2X receptor sequences of both a fungus (*G. margarita*) and an amoeba (*D. discoideum*) but their absence from the invertebrate-only group suggests that the second group of P2X receptors may actually be phylogenetically distinct purinergic receptors.

### Structural homology of P2X receptors

Further analysis of the receptors by 3D protein modelling shows that sponge P2X receptors are nearly identical in structure to the zebrafish P2X4 subunit (Fig. 4C), of which the crystal structure is known and depicted as a ‘dolphin’ with extensive folding to create a ‘head’ region with the N- and C-terminal helices creating a ‘tail’ (Kawate et al., 2009). The structural similarity of the two *E. muelleri* P2X receptors suggests that while there is little amino acid sequence conservation, preserving the structure of the protein is more important to its function, which Leys and Riesgo (2011) also concluded when investigating type IV collagen in sponges. However, these differences would still affect the biochemistry of the receptor: the P2X receptor of *A. californica*, despite having a similar structure to that of *L. stagnalis*, still had noticeable differences in amino acid sequences in key functional domains, and, as such, had different pharmacological and kinetic properties (Györi et al., 2021). This may explain why sponges were sensitive to apyrase, which may have disrupted endogenous ATP signalling, and why ADP, which is not usually a P2X receptor ligand, was able to trigger sneezes from sponges.

Certain regions and amino acid residues in the set of P2X receptor sequences we analysed are highly conserved, unlike the conserved motifs that were absent in a subset of P2X receptors (Fig. S2). These are likely to be domains and specific residues that are critical for purinoceptor function. While P2X receptor subtypes themselves have different affinities for ATP (Hou and Cao, 2016), there is very little activation by ADP because of the positioning of positively charged residues within the ATP binding site (Chataigneau et al., 2013). Results from our physiological experiments showed that ADP was sufficient to trigger contractions in sponges (Fig. 3B). It is possible that receptors encoded by the Em0004g666a gene (and others in its group) are not as specific as conventional P2X receptors for ligand binding. Differences in the sequence and structure may therefore allow activation by ADP, although this hypothesis requires further investigation.

### Conclusions

The present study demonstrates that sponges use purinergic signalling to coordinate behaviour. We provide evidence that ATP signalling is necessary for contractions in sponges to occur, and that sponges respond to ATP in a concentration-dependent manner. Interestingly, constant exposure to excess ATP in the bath application causes a sustained dilation of the excurrent canals, preventing the completion of the sneeze ‘expansion–contraction’ cycle, which suggests that normally there must be a mechanism for removing ATP from the extracellular space to prevent over-activation of the sponge tissues. Our finding that glutamate and ATP signalling work in concert to coordinate contractions of the sponge and the conservation of P2X receptors from sponges demonstrate an interesting parallel between signalling pathways involved in coordinating sponge behaviour and signalling networks used in animal nervous systems.

## Supplementary Material

10.1242/jexbio.248010_sup1 Supplementary information

Table S2. Supplementary Excel file with species names, abbreviations, and NCBI reference numbers used in bioinformatic analysis.

Dataset 1. Supplemental Excel file with experimental data. Excel sheet tabs are titled according to the experiments.
