# Supplementary material for: ATP and glutamate coordinate contractions in the freshwater sponge Ephydatia muelleri
Source: J Exp Biol. 2025 Feb 12;228(3):JEB248010. doi: 10.1242/jeb.248010 (PMC11883242; doi:10.1242/jeb.248010)
Supplement: Supplementary information [file jexbio-228-248010-s1.pdf]

>mus_1
MARRLQDELSAFFFEYDTPRMVLVRNKKVGVIFRLIQLVVLVYVIGWVFVYEKGYQTSSGLISSV
SVKLKGLAVTQLQGLGPQVWDVADYVFPAHGDSSFVVMTNFIMTPQQAQGHCAENPEGGICQD
DSGCTPGKAERKAQGIRTGNCVPFNGTVKTCEIFGWCPVEVDDKIPSPALLHEAENFTLFIKNSIS
FPRFKVNRRNLVEEVNGTYMKKCLYHKILHPLCPVFSLGYVVRESGQDFRSLAEKGGVVGITID
WECDLDWHVRHCKPIYQFHGLYGEKNLSPGFNFRFARHFVQNGTNRRHLFKVFGIRFDILVDGK
AGKFDIIPTMTTIGSGIGIFGVATVLCDLLLLHILPKRHYYKQKKFKYAEDMGPGEGERDPAATSS
TLGLQENMRTS
>mus_2d
MAAAQPRLPAGAAMVRRLARGCWSAFWDYETPKVIVVRNRRLGFVHRMVQLLILLYFVWVAS
GAGTALSHRYVFIVQKSYQDSETGPESSIITKVKGITMSEHKVWDVEEYVKPPEGGSVVSIITRIEV
TPSQTLGTCPESMRVHSSTCHLDDDCVAGQLDMQGNGIRTGRCVPYYHGDSKTCEVSAWCPVE
DGTSENHFLGKMAPNFTILIKNSIHYPKFKFSKGNIASQKSDYLKHCTFDQDSDPYCPIFRLGFIVE
QAGENFTELAHKGGVIGVIINWNCDLDLSESECNPKYSFRRLDPKYDPASSGYNFRFAKYYKING
TTTTRTLIKAYGIRIDVIVHGQAGKFSLIPTIINLATALTSIGVGSFLCDWILLTFMNKNKLYSHKKF
DKVRTPRHPSSRWPVTLALVLGQIPPPPSHYSQDQPPSLPSGEGPALGEGAELPLAVQPPRSCSSS
ALTEQVVDTLDQHMGQRPPVPEPSQQDSTSTDPKGLAQL
>mus_3
MNCISDFFTYETTKSVVVKSWTIGIINRAVQLLIISYFVGWVFLHEKAYQVRDTAIESSVVTKVKG
FGRYANRVMDVSDYVTPPQGTSVFVIITKMIVTENQMQGFCPENEEKYRCVSDSQCGPERFPGG
GILTGRCVNYSSVRRTCEIQGWCPTEVDTVEMPIMMEAENFTIFIKNSIRFPLFNFEKGNLLPNLTD
KDIKKCRFHPEKAPFCPILRVGDVVKFAGQDFAKLARTGGVLGIKIGWVCDLDKAWDQCIPKYS
FTRLDGVSEKSSVSPGYNFRFAKYYKMENGSEYRTLLKAFGIRFDVLVYGNAGKFNIIPTIISSVA
AFTSVGVGTVLCDIILLNFLKGADHYKARKFEEVTETTLKGTASTNPVFTSDQATVEKQSTDSGA
YSIGH
>mus_4a
MAGCCSVLRAFLFEYDTPRIVLIRSRKVGLMNRVVQLLILAYVIGWVFVWEKGYQETDSVVSSV
TTKAKGVAVTNTSQLGFRIWDVADYVVPAQEENSLFIMTNMIVTVNQTQGTCPEIPDKTSICDSD
ANCTLGSSDTHSSGIGTGRCVPFNASVKTCEVAAWCPVENDAGVPTPAFLKAAENFTLLVKNNI
WYPKFNFSKRNILPNITTSYLKSCIYNARTDPFCPIFRLGQIVADAGHSFQEMAVEGGIMGIQIKW
DCNLDRAASHCLPRYSFRRLDTRDLEHNVSPGYNFRFAKYYRDLAGNEQRTLTKAYGIRFDIIVF
GKAGKFDIIPTMINVGSGLALLGVATVLCDVIVLYCMKKRYYYRDKKYKYVEDYEQGLSGETD
Q
>mus_5a
MGQAAWKGFVLSLFDYKTAKFVVAKSKKVGLLYRVLQLTILLYLLIWVFLIKKSYQDIDTSLQS
AVVTKVKGVAYTNTTMLGERLWDVADFVIPSQGENVFFVVTNLIVTPNQRQGICAEREGIPDGE
CSEDTDCHAGESVVAGHGLKTGRCLRVGNSTRGTCEIFAWCPVETKSMPTDPLLKDAEGFTIFIK
NFIRFPKFNFSKANVLETGNKHFLKTCHFSSTNLYCPIFRLGSIVRWAGADFQDIALKGGVIGIHIE
WDCDLDKAASHCNPHYYFNRLDNKHTQSISSGYNFRFARYYRDPHGVEFRDLMKAYGIRFDVI
VNGKAGKFSIIPTVINIGSGLALMGAGAFFCDLVLIYLIRKSEFYRDKKFEKVRGQKEEDNVEVEA
NEMEQELPEDKPLERVHQDEQALELAQSGRKQNSNCQVLFEPARSGLQENAFVNMKPSQILQTV
KT
>mus_6a
MQLQPAGTGNMASAAAAALVSWGFLDYKTEKYVLTRNCRVGVSQRLLQLAVVVYVIGWALL
AKKGYQERDLAPQTSVITKLKGVSVTQVKELENRLWDVADFVKPSQGENVFFLVTNFLVTPAQ
VQGRCPEHPSVPLANCWADEDCPEGETGTYSHGIKTGQCVVFNGTHRTCEIWSWCPVESGAVPR
KPLLAQAKNFTLFIKNTVTFSKFNFSRSNALLTWDNTYFKHCLYDPLSSPYCPVFRIGDLVAMAG
GDFEDLALLGGAVGISIHWDCNLDTKGSDCCPQYSFQLQQKGYNFRTANHWWAASGVETRSLL
KLYGIRFDILVTGQAGKFALIPTAITVGTGAAWLGMVTFLCDLLLLYVDREAGFYWRTKYEEAR
APKTTTNSS
>mus_7a

Journal of Experimental Biology • Supplementary information

MPACCSWNDVLQYETNKVTRIQSTNYGTVKWVLHMIVFSYISFALVSDKLYQRKEPVISSVHTK
VKGIAEVTENVTEGGVTKLGHSIFDTADYTFPLQGNSFFVMTNYVKSEGQVQTLCPEYPRRGAQ
CSSDRRCKKGWMDPQSKGIQTGRCVPYDKTRKTCEVSAWCPTEEEKEAPRPALLRSAENFTVLI
KNNIHFPGHNYTTRNILPTMNGSCTFHKTWDPQCSIFRLGDIFQEAGENFTEVAVQGGIMGIEIYW
DCNLDSWSHHCRPRYSFRRLDDKNTDESFVPGYNFRYAKYYKENNVEKRTLIKAFGIRFDILVFG
TGGKFDIIQLVVYIGSTLSYFGLATVCIDLLINTYSSAFCRSGVYPYCKCCEPCTVNEYYYRKKCE
SIMEPKPTLKYVSFVDEPHIRMVDQQLLGKSLQVVKGQEVPRPQMDFSDLSRLSLSLHDSPLTPG
QSEEIQLLHEEVAPKSGDSPSWCQCGNCLPSRLPEQRRALEELCCRRKPGRCITTSKLFHKLVLSR
DTLQLLLLYQDPLLVLGEEATNSRLRHRAYRCYATWRFGSQDMADFAILPSCCRWRIRKEFPKT
EGQYSGFKYPY
>human_1
MARRFQEELAAFLFEYDTPRMVLVRNKKVGVIFRLIQLVVLVYVIGWVFLYEKGYQTSSGLISSV
SVKLKGLAVTQLPGLGPQVWDVADYVFPAQGDNSFVVMTNFIVTPKQTQGYCAEHPEGGICKE
DSGCTPGKAKRKAQGIRTGKCVAFNDTVKTCEIFGWCPVEVDDDIPRPALLREAENFTLFIKNSIS
FPRFKVNRRNLVEEVNAAHMKTCLFHKTLHPLCPVFQLGYVVQESGQNFSTLAEKGGVVGITID
WHCDLDWHVRHCRPIYEFHGLYEEKNLSPGFNFRFARHFVENGTNYRHLFKVFGIRFDILVDGK
AGKFDIIPTMTTIGSGIGIFGVATVLCDLLLLHILPKRHYYKQKKFKYAEDMGPGAAERDLAATSS
TLGLQENMRTS
>human_2.d
MAAAQPKYPAGATARRLARGCWSALWDYETPKVIVVRNRRLGVLYRAVQLLILLYFVWYVFIV
QKSYQESETGPESSIITKVKGITTSEHKVWDVEEYVKPPEGGSVFSIITRVEATHSQTQGTCPESIR
VHNATCLSDADCVAGELDMLGNGLRTGRCVPYYQGPSKTCEVFGWCPVEDGASVSQFLGTMA
PNFTILIKNSIHYPKFHFSKGNIADRTDGYLKRCTFHEASDLYCPIFKLGFIVEKAGESFTELAHKG
GVIGVIINWDCDLDLPASECNPKYSFRRLDPKHVPASSGYNFRFAKYYKINGTTTRTLIKAYGIRI
DVIVHGQAGKFSLIPTIINLATALTSVGVVRNPLWGPSGCGGSTRPLHTGLCWPQGSFLCDWILLT
FMNKNKVYSHKKFDKVCTPSHPSGSWPVTLARVLGQAPPEPGHRSEDQHPSPPSGQEGQQGAEC
GPAFPPLRPCPISAPSEQMVDTPASEPAQASTPTDPKGLAQL
>human_3
MNCISDFFTYETTKSVVVKSWTIGIINRVVQLLIISYFVGWVFLHEKAYQVRDTAIESSVVTKVKG
SGLYANRVMDVSDYVTPPQGTSVFVIITKMIVTENQMQGFCPESEEKYRCVSDSQCGPERLPGG
GILTGRCVNYSSVLRTCEIQGWCPTEVDTVETPIMMEAENFTIFIKNSIRFPLFNFEKGNLLPNLTA
RDMKTCRFHPDKDPFCPILRVGDVVKFAGQDFAKLARTGGVLGIKIGWVCDLDKAWDQCIPKY
SFTRLDSVSEKSSVSPGYNFRFAKYYKMENGSEYRTLLKAFGIRFDVLVYGNAGKFNIIPTIISSVA
AFTSVGVGTVLCDIILLNFLKGADQYKAKKFEEVNETTLKIAALTNPVYPSDQTTAEKQSTDSGA
FSIGH
>human_4.1
MAGCCAALAAFLFEYDTPRIVLIRSRKVGLMNRAVQLLILAYVIGCYHPHLAEVEMESPRRWVF
VWEKGYQETDSVVSSVTTKVKGVAVTNTSKLGFRIWDVADYVIPAQEENSLFVMTNVILTMNQ
TQGLCPEIPDATTVCKSDASCTAGSAGTHSNGVSTGRCVAFNGSVKTCEVAAWCPVEDDTHVPQ
PAFLKAAENFTLLVKNNIWYPKFNFSKRNILPNITTTYLKSCIYDAKTDPFCPIFRLGKIVENAGHS
FQDMAVEGGIMGIQVNWDCNLDRAASLCLPRYSFRRLDTRDVEHNVSPGYNFRFAKYYRDLAG
NEQRTLIKAYGIRFDIIVFGKAGKFDIIPTMINIGSGLALLGMATVLCDIIVLYCMKKRLYYREKKY
KYVEDYEQGLASELDQ
>human_5.a
MGQAGCKGLCLSLFDYKTEKYVIAKNKKVGLLYRLLQASILAYLVVWVFLIKKGYQDVDTSLQ
SAVITKVKGVAFTNTSDLGQRIWDVADYVIPAQGENVFFVVTNLIVTPNQRQNVCAENEGIPDG
ACSKDSDCHAGEAVTAGNGVKTGRCLRRENLARGTCEIFAWCPLETSSRPEEPFLKEAEDFTIFIK
NHIRFPKFNFSKSNVMDVKDRSFLKSCHFGPKNHYCPIFRLGSVIRWAGSDFQDIALEGGVIGINIE
WNCDLDKAASECHPHYSFSRLDNKLSKSVSSGYNFRFARYYRDAAGVEFRTLMKAYGIRFDVM
VNGKGAFFCDLVLIYLIKKREFYRDKKYEEVRGLEDSSQEAEDEASGLGLSEQLTSGPGLLGMPE
QQELQEPPEAKRGSSSQKGNGSVCPQLLEPHRST

Journal of Experimental Biology • Supplementary information

>human_6.1
MCPQLAGAGSMGSPGATTGWGLLDYKTEKYVMTRNWRVGALQRLLQFGIVVYVVGWALLAK
KGYQERDLEPQFSIITKLKGVSVTQIKELGNRLWDVADFVKPPQGENVFFLVTNFLVTPAQVQGR
CPEHPSVPLANCWVDEDCPEGEGGTHSHGVKTGQCVVFNGTHRTCEIWSWCPVESGVVPSRPLL
AQAQNFTLFIKNTVTFSKFNFSKSNALETWDPTYFKHCRYEPQFSPYCPVFRIGDLVAKAGGTFE
DLALLGGSVGIRVHWDCDLDTGDSGCWPHYSFQLQEKSYNFRTATHWWEQPGVEARTLLKLY
GIRFDILVTGQAGKFGLIPTAVTLGTGAAWLGVVTFFCDLLLLYVDREAHFYWRTKYEEAKAPK
ATANSVWRELALASQARLAECLRRSSAPAPTATAAGSQTQTPGWPCPSSDTHLPTHSGSL
>human_7
MPACCSCSDVFQYETNKVTRIQSMNYGTIKWFFHVIIFSYVCFALVSDKLYQRKEPVISSVHTKV
KGIAEVKEEIVENGVKKLVHSVFDTADYTFPLQGNSFFVMTNFLKTEGQEQRLCPEYPTRRTLCS
SDRGCKKGWMDPQSKGIQTGRCVVYEGNQKTCEVSAWCPIEAVEEAPRPALLNSAENFTVLIKN
NIDFPGHNYTTRNILPGLNITCTFHKTQNPQCPIFRLGDIFRETGDNFSDVAIQGGIMGIEIYWDCN
LDRWFHHCRPKYSFRRLDDKTTNVSLYPGYNFRYAKYYKENNVEKRTLIKVFGIRFDILVFGTG
GKFDIIQLVVYIGSTLSYFGLAAVFIDFLIDTYSSNCCRSHIYPWCKCCQPCVVNEYYYRKKCESIV
EPKPTLKYVSFVDESHIRMVNQQLLGRSLQDVKGQEVPRPAMDFTDLSRLPLALHDTPPIPGQPE
EIQLLRKEATPRSRDSPVWCQCGSCLPSQLPESHRCLEELCCRKKPGACITTSELFRKLVLSRHVL
QFLLLYQEPLLALDVDSTNSRLRHCAYRCYATWRFGSQDMADFAILPSCCRWRIRKEFPKSEGQ
YSGFKSPY
>gallus_2a
MGPGCRRAVSSYSSPQVVVVRDGRLGTAYRALQLLVLLYFIGYVFIVQKGYQERETGPESSVITK
VKGVTQSLSKVWDVGEYVAPPEGGSSFSILTRVEVSAAQAMGTCHEGPSAACHSEQDCVSGAM
DASHHGVRTGRCVPGGSGRSCEVLAWCPLHGGSSSESLAEMAAQFTILIKNHVRFPRFGFSKANI
QAAESHYLKSCTFNATSALYCPIFRLGFLAEQAGEDFAVLAEKGGVIGVIISWDCNLDLPDTECN
PRYSFRRLDPKGALASPGYNYSPHPCACMPPRFAKYYSWNGTCTRVLTKAYGIRVDVIVQGQAG
KFSLIPTVITLATALTSVGLGSFLCDWVLLCCMDKERRYSSRKFEQVPLG
>gallus_3
MPPPRPQPRCLADFFSYETTKSVVVKSWVVGVVNRVVQLLILSYFIGWVFLHEKAYQVRDTVIE
SSVVTKVKGIGKYGNRVLDTADYVTPPQGTSVFVVVTKQILTENQEQGVCPESEAAYRCASNRD
CQGKGRTTGSGVLTGRCVPYNVTLHTCEIRGWCPPEVDTVDVPVMLEAENFTLFIKNSVRFPLFG
FEKANLPPQVSAGELQRCRFHPEQQPLCPILRLGDVARFAGQDFASLAATGGVLGIKIGWVCDLD
RAWELCLPRYSFTRLDSVTQHSPGSPGYNFRHARYYRGHNGTELRTLTKAFGIRFDVLVYGNAG
KFGIVPTLINTVAAFTSIGVGTVLCDIILLNFLKGAEHYKARKFEEVPEASVSPAPTSPTVCAPGAL
GDQSREKQSTDSGTFSLGL
>gallus_4a
MAACCGAVRGFLFEYDTPRIVLIRSRKVGLINRAVQLAILAYVIGWVFLWEKGYQETDSVVSSV
TTKVKGVTMTNTSALGSRIWDVADYVIPPQEKNAVFVMTNMIFTLNQSQSHCPELPDDNTECNN
NSSCVPGYVSTHSNGIQTGACIPYNSSIKTCEVFAWCPVEDDYHIPNPAFLQGAENFTILVKNNIW
YPKFNFSKRNILPTFSSSYLKNCIHDAQTDPFCPIFRLGKIVEAAGQNFQEMAVEGGVMGLQINW
DCNLDRAASHCVPKYSFRRLDNKDSANTISPGYNFRFAKYYKDSSGIETRTLIKAYGIRLDIIVFG
KAGKFDVIPTMINIGSGLALFGVATVLCDIVVLYCMKKRYFYREKKYKYVEDYELGTSET
>gallus_6a
MATPAGGGAALCGPLLDYKTEKFALTRNRRVGLLHRLLQLAVLGYVLGWVFVVRRGYQDTDA
APRVSVVTKLKGVSVSRPEDARRRLWDAADFSRPPQGENVLFLVTNFIVTDRQVQGTCPESPSVL
DGICMEDVDCSVGNPVVHGNGIKTGRCVMFNSTHSTCEIFGWCPVENDTLPRKPLLAEAENFTL
FIKNTVNFTKFNFSKGNTLQTNDPTYFKTCMYDPFFSPSCPVFRIRDMVEAAGETFGALALMGGS
ISVRIEWDCDLDWPAAHCQPRYSFILLDRRYNFRPPSSVTWFCSTWMRRLTSIGRRSLRRQNLPK
VRKQQQLRHRGLARTWGMLSNAHWSDLQECQHQQKSLVHREYMESPPDPTCSSRAMLQSCCQ
GWGSLGWGRFTLSTRSLKHFALLPFPLGPVKSRDALK
>gallus_7

Journal of Experimental Biology • Supplementary information

MVAWGWMKDVFNYESPKLIRFPSVGLVCVKWFIYGVIAVYICYTLIVHKRYQEKEELTSSVRVT
LKGVARVDRIWDAAEYTIPTQTRDSFFVMTNIIRTENQIQKTCPEYPTAKAICSSDKSCAKGIVDV
HSNGVQTGKCVHYNITHKTCEIKAWCPVQGEERPPVPAVLRSSEDFTVFIKNNIHFPTFNYTVQNI
SPKLNTSCKFNKVTAPLCPIFRLGDILQEAKENFSEMAVKGGIIAIEIKWDCDLDSWSYYCSPEYS
FRRLDDKTRTQYPGFSIRFARHYKLPDGTEQRTLFKAYGIRFDVLVFGMGGQFKLIELFTFIGSTIA
YFGLAVTIIEMCFHLYNCSSCCKIQVCENVIRKKYETVLMPEQVILVSYVDKPHITLIKMPLRTSL
QNAEGSIFEDHPVKSYDPRTCCSHKSNEKHGAAQSELRPLTQSSSSTNCPKWCCCGRCQVAQKH
HEQLCCRKKEGQCITTTYWFAQLVLSRDTLNKALLYEDPFLDLTGHSSNSQLRRIAYKQYIHWR
FGSFELEDRAIIPSCCRRLIRSTYPKENGNYTGFNLE
>xenopus_4
MSRDGCCGQAYSCLFDYDTPRIALIKSRKIGLLNRFIQLGILAYVIGWVFIWEKGYQEFDIVVSSV
TSKVKGVVVTNTTELGVKIWDVADYIIPAQEENAVFVMTNLILTQNQTQGHCPELPETSFCSKEQ
PCTPGYVGKQSNGVQTGKCVPYNSTVKTCEIFAWCPVENDTHVPDPAFLNGAENFTVLIKNNIW
YPKFQVSKRNILSNISSSYLKTCQYDKVNHPFCPIFRLGNIVKEAGESFSDMAVQGGVMGIQINW
NCDLDRKLTYCVPKYSFRRLDNREIDHNVSPGYNFRFAKYYKDSNGVESRTLMKVYGIRFDILV
FGTAGKFDIIPTMINIGSGAALFGVATVLCDMIVFHFFKKRHYYREKKYKYVEDYDELVGSECGS
NP
>xenopus_6a
MDAGCAQAQPALLDYKTEKYLLTKNRKVGVIYRFLHLAILCYIIGWVFLAKKGYQDTDSDPHFS
IITKLKGVSITKNFRNSQEKIWDVADFVKPSQGENVLFLVTNFIATTGQVQRTCAENPVNSDARCS
ENLDCVAGEPVVNGNGIKTGKCVMFNHTQSTCEIYGWCPVENESLLRKPRLNEAENFTLFIKNV
VHFSKFNFSRANTLDTSDETYFKNCRYNPVSSPYCPVFQIQEIITQAGQSFEELSVMGGVVAARIE
WKCDLDRPAAECLPQYSFRLQDTKNNFRTATYYWDQERKEYRDLFKLYGFRFDISVTGEARKF
GVVPTAVSLGTGCAFLGAATFLCDLILLYLDKKASFYRSCKYEEVKPPKNQQTIQETRNE
>xenopus_7
MESSGSESQSNQSESSVCLDTDPLCDNLVRQLQQSLGDCLNDNCFPDDPVWPPGEREKRMEQRQ
HPRLGNTLWCLCGNCIAMPTIRESVCCREVEKLQKHYNEDCTCITTVLDMLQLCINKHFLEFTIR
NSGRVKLRQLQDDYNRCMRKGAYRSFTTWTHGILGPKTRIPIPSCVVRLVRNNYPDPHGHYVGF
REATDNPAEDMAFEDTF
>danio_3b
MQQCSGKMWTCITDFFTYETTKSVVVKSWTIGIINRVVQLLIITYFIGWVFLYEKAYQVRDTAIES
SVMTKVKGFGEYNNRIMDVADYVTPTQGASVFCVITKLITTENQVQGNCPESELKFKCENDIRC
KQLMTKPSSNGLLTGRCVHFNTTHKTCEIKGWCPAEIDEVQPNPMMEVENFTIFIKNSIRFPRFNF
TKGNFLANINSSYIKGCNFDFERNMYCPIFKVGDVIRFAQQNFTTLATKGGVIGIKIAWVCDLDK
ADDECKPAYSFTRLDAMSEKTTVSPGYNFRYAKYFKMENGTEYRTLLKAYAIRFDVLVNGDAG
KFDMIPTLINMVAAFTSVGVGTVLCDIILLNFLKGADQYKAKKFEEVSDSNIQSSGSLYRTRDHS
QQSIKTDEKFSNDSGAFSIERYS
>danio_4b
MGCSAYCFHCLFDHGTPVILVVSSKKVGFIFRLLQLCIIVYVAVYVCWYKRAYQERDSVISSVST
KVKGNILTNSSAFGTHVWDTSEYVIPPQGENSFFVLTNAIVTQGQTQGSCPEIPSEFTNCQSDSDC
KEGLKEIRGNGVQTGKCVQYSETIKTCEVLSWCPLENDTVIPKPALLSAAEDFTVLIKNNIQYPKF
KFRKRNILRHINSTYLKNCTFNHKTDPHCPVFRLGDIVTESGEDFSIMALKGGIIGIFIDWSCDLDF
NERFCVPKYSFRRLDNKNPENNVAPGYNFRYAKYYMNNENTETRTLIKAFGIRFDVIVFGMAGK
FNIVPTIVNVGAALALLNLMKVICDWFMLNCLSDSEYYSKHKFKHLKPKDESETFSFTDQSSYGT
P
>danio_5
MAQTWGNFFFSLLDYKTEKFVIAKNKKVGVLFRLFQLTVIGYLIGWVFIWKKGYQETEEAIQSS
VITKLKGVDLTNSSQFGLQLWGAEDYVIPPQGDRVFFVVTNYLVTPNQRLGYCPESPKVPDGFC
TNDNECVEGESVLAGHGVKTGRCLNDTGTCEINAWCPVEHGHAPVEPMLAKAENFTVYVKNFI
KFPKFGFFKSNVLPITNSTYLKTCRYDKDHHPYCPIFLVGDVINWTGYTFQDLATRGGSIGIGIEW
NCDLDKDESHCNPEYSFTRLDSSENSQHSVTSGYNFRFARYYNDAAGQTYRNLFKVYGIRFDILV

Journal of Experimental Biology • Supplementary information

NGKAGRFSIIPTVINIGSGLALMGAGVFACDMILLYMMSKSSFYRETKFEAIKKKQSERESREQRE
RKHARHHRHHHHHRQDGRHRREEKPTAEMQPLTSILTQANNNPETQTQPSAASALKPHSPERKP
QATVSFRTHTTEKHTVSPVSTLRAARHQ
>danio_7
MPCVLLNLCEYDTQKLVKIKSVKLGSLKWTLNGVILMFICIMMLWNKEYQEYDFVVSSVTTKV
KGVAKITLPEVGDVVWDVVDYSGPSQGKNSFFVATNAIVTKNQKQGNCAEILPNGKLCRTDKD
CEKGFSDQHSHGVQTGACVKLEILKKTCEVTAWCPIENKKNPRPALLAAAENFTVMIKNNIRFPA
FNYIRRNILSEMKDTDFKGCIYHRYKNPYCPIFRLGDIVAEAKEKFSEMAVEGGVIGIQINWDCDL
NRFFHSCLPKYSNRRLDEKESNRTLYPGLNFRFARYSTVNGVEQRTLFKMYGIRFDVMVFGKAG
KFSIIQLIIYIGSTLSYYAITTIFLDWLIGTGYYSKEAKQNYTERKFEAVQDREECFLCVSFVDEDNL
RVVKKSRKKILQETKPLSIHQRKNELASMKTLLSVLQCGQSRSEPVQNGQSGGLIVDENLSSRHN
GRQNPDTPLLETTQSSSPTWCQCGSCRPAETLQEQLCCRLKKGRCITSSPIFSSLIVSRSVLENALF
FVDPLAELHEESQLRHGAYAQFIRWRFGDSTPRDALPVIPSCCIWRIRAEYPSPDGTYRGLRSFQV
ITSQTEVNR
>ciona_07
MEGDDSFEGLSDLSSSLDLVCEITESDSDDSEVSGSIKPYRYEPESNEQTEESVMSPEATLTVDRR
QNTNWCQCGLCECMAAAQESVCCREQEQVSQKMPDTANCITEHKNFDPVCLNEDVLEVAYST
YQQHSNLNDGNNWKRYTAYRQFIRWCYGYLGMKVRVPIPACVVNKIRHSFPLQEGQEYVSFRF
AD
>spur_4
MLAVGTNDVGLLTFAMPVHESLSRGIATVFDKYVIFWQKGYQSTDNVEGSATSKLKGVAYTNIS
NSSDLRTPFPERYTRVWDVSDYVIPAQQTNGFFVMTNMVLSPMQSRGACPEDPEIDRDACTTDV
ECKPDEGIITGNGIKTGRCVNATHPKTKEATTVCEVKAWCPVEVDIRPLWTSAILEEAENFTVLIK
NSISFPKFGFVKRNILDTTDPMYLRSCRYSRLKDPLCPIFRLGTIVAETGQSFRQMAIKGGVITIDIQ
WNCNLDLSYKLCLPKYRFIRADEQDAKIAGGFNFSWFDTPLRVGFGKFYRINDTEYRDLTKAYG
ILFQVKITGVAGKFDIVPLMLNFASGVALLSLATVMCDVVVLYLLKKRKYYKEAKFQNVASNEN
GPQKTYEEVAALCEQALVTSLPITQLNNNHQTPPGMGGSNHSLRDEITHPTTNETKKPTSL
>spur_7
MSSSDEPVCYGFDPEYSEAELAEMEERRAAIDQEVDVVREDAPDPLPAPDRMENNDWCLCGNC
PVMPTVVECVCCRELARCRELQPEGCVIDAREFGEVCLTVAVLRVFHIARRDIRGHRRHVPGELE
NISYRFAGYKLFTYLVHGYLGKGVRRVVPACATTRIRLEFPEDPAVYVGFKYGDDGEIMEVPEQ
LAI
>hydra_E
MSLFHYITYKCIIIKDKRLGFAYYSLALIILLYTFVQIIINKAYLKFDYSPKGSIRLLVSTPPFNNAAH
PSYCDNNLCKRVDGLTLNWPVESKAVTIATFMKEIVQEKKCFTNSINDINFNTHTDYSAISHSSY
YPMYPEEALVKIEHSISTSSLFSASHRMMTGVLLGSDGHPLKFLNSNNSSSVDKLYLKDFLKAAN
ISLDRHSDALSSRNRTYRQNGLVLRVTIDYSNAKGWFGKGKTEYTYSVEHNLYSDYRIKQELPIA
LPNGTICKRVVIKRYAVRIEFVQVGKIGEYSMSNLILQIVSLMGLLTLATTIIDVAALYVVPDKGIY
RQYVFDSSPELQTNVRDEKANLLEKKDQ
>hydra_4a
MANDCCKNFSKILFHYETVKIVDIKNKKVGALYRFIQLIILAYVIGYVIVYKKGYQDFDNAISTVT
TKLKGTSYVNYNNVTSPFHENIEVYDPADYVVPPQENNAFFVMTNMIITPNQTRSRCPEDPKFSE
NKCQVDKDCLPALKPIKNGNGVRTGRCVQSDRPNISYRVCEIYGWCPTETDILPMPGYNFSASVP
LLDDAKEFTVLLKNEVIFPKFKVQRRNIVKNNNSNYLKTCFHNHDTDPLCPIFKIKDVIQECNDDF
EQVAYLGAVYGILVNWDCNLDLSLDKCTPTYSTRRLDDPDAPISPGFNFRFANYYVVDNKQYRT
LFKAYGLKFEIIVTGKAGKFSVIPLFTNLGAGLALLGIAVVWCDFIVMYLLKKRFIYKEYKYQKID
DDEETNKNEAAEEVGNQIKEKSESKVGYQLLDDEDNH
>nvec_E1
MSLLHYITYKLVVIRDKRLGTIYYTFVAAIILFTLVEIFVFKGYLEYDTSPEGTMRILVSDPKDDND
NHPVIKTHVPSYCCKGNANRTCSLCEYIDAQELSWPIESHTINLMTFGKDRWQVKQRNDSFLKQ
EFVNIREKKYFTVNPEEVMIKVEHSIVATRFTRKSGKEQVAASQRSMKGYLYDAQGKIIQSIPAT

Journal of Experimental Biology • Supplementary information

NFKKPGLGQADKLTVQELLNAAGIDSLEQPSDALNAKGKSFRRHGMVLHVGICYHNTETTLIGT
GNIEYSYHVRRIPYADYRINQVIPIIGADDFSFTDDKTLTRPEKRLFRKRYGIKIEFHQSGKLGRFSL
PALLLKLVSGVGLLTLTATIVDTAALYLLPDRFQYREFVYEESPVIDKRRCKGGEQRSDETATKS
KKES
>nvec_4a
MAPSCGNCCACVGSVLFEYDTEKIVHIKNKKIGIINRVIQLVIIAYIIGFVIVYKKGYQEFESPYSSV
TTKLKGVALTNLTGANLSYYGREFLWDSADLVIPPEEEGAVFMMTNMIITPEQSQGKCPEDPSVP
GARCTADADCPPLQSTSTSHGVNTGNCVPSTHNMTVKTCELYAWCPIENDVTPMPGFNLSYGM
PLLDAAKNFTLLIKNSVQFPKFSESRRNIIQKNDSHLKSCNYHPTSDPLCPVFRLGTMAELAGVNF
EKLAYKGGVMAIVITWNCNFDPLAYHCEPKYSFRRLDDDESPIAPGYNFRFPRYYIKNETLYRTI
VKAYGIRFVVTVYGKGGKFSVIPLFLNIGSGLALLGIATVLCDVFVLYVLRKKYFYREKKYLNVD
DSDNEMSLFQKADKVHSKRGYSQLSDNEGEMMK
>nvec_7
MDFIDSLLVMEDDDNDREEENFGLAMEPMDVAGPSTHPQGPTAVGITPAPRPPGEPLPDWCKCG
NCREMPQQIENVCCGKRNCESTKARFGKLCLDVEVLSLGIRSSADIRNDRHDSSARAFRKAGYR
NYILDKHGYLGKGRRRVPPSCIVWQIRHHYPTKTGIYMGFREN
>acropora_E
MSFFHYITYKLVIIRDRRIGFIYYSIAAAIVLYTFAEIFLKKGYLELDTSPEATLRFALSDPKEENGG
NESGQIPSFENLTYCCNRDVEPSKLCTPCQFLDAQELSWPVESHTISLTTFAKDRWQMRNTSFSS
MSNPTQFETIKESLYFTAGPESMLVKIEHAVLATKLFSGGTDLAASQRKMKGYLIGHDGKVLRSL
SADENMNPRGRADRLTIQEILNAAGVAGLDEVSDALNAKGKPFRRHGIVLHVTIHYQNTDSTILG
TGDITYSYHVRRIPYADYRVNQLMPVRSEKEFAADSSHRTHQENRLFRKRYGIKIEFHQSGRLGH
FSLPALLMRLVSGVGLLTLTATIVDILTLYILPDKFRYRRFVYEESPVLELKKNE
>acropora_4a
MVACDEFCSAVLAVFFEYDTNKIVHIKSKKVGLINRFIQLVIIVYIIGYVIVYKKGYQEFQEPYSSV
TTKVKGVSLTNLSQQLALYGGVHVWDSSDFVIPPEEDNAVFVMTNMIISPNQTQSTCPEDPKFPN
VKCTKDSDCPPLKPVENGHGLRTGRCVLSDREKNKKVCEIYSWCPVEIDELPMPGNNLSKNMPL
LGAAKDFTLLVKNNVQFPKFQETARNIKSAGNTSYLKGCHYDPKTDPLCPIFKFDKMFELANITF
EDIAFQGGVMAIIIKWNCNFDPLAYSCEPKYQFTRLDDSKSPIAPGYNFRFAKYYVKDNVLYRTL
LKAYGIRFAILVYGEGGKFSPVPLFLNLGSGLALLGVATVLCDIVVLYVLQKKYFYREKKYQYV
EDVESNYFPGYQKSNNLHSANSMDVDNNQYSSFTNELPKEDIVQ
>acropora_7
MERITCSCRGQCALKRGRGACVCKREDLKCVASCKCDKSKCKNKDNTLERNQRAAEATPTSGA
EAFAASIERTRQEIREFVESLDRDQQVELLCKLLSMGRGSLEFAKHAVAQGIPEQPSPLEEQPPWC
QCHICRPMESEQENLCCKKRTCITSYTSFSNICLDREILEVCIKARCDIRAEEFNFTMESFRKAAYR
QYVLWTYGKLGRGNRRVIPACVVRMIRAAYPAPDGQYMGFKNN
>daphnia_4a
MVIYFEIIQVLNQHLINVPCWYNLRSCNVSLTMGCFKSMSSFLFWYETPKVVHIRSKKVGILGRF
LQLCVLSYIVGYVMVYKKGYQEFCSVESAVVTKVKGVAFTNTQRNVPEIYKRIWDTSDLIVPPS
ENDAFFVTTNIIITPNQTRRTCSEDPGVHGALCNTPSQCHEGHSLPIGNGAMTGRCVPSDVNSSVN
VCEIFAWCPIEQDVFPLGLDRPLLEETADFTVLIKNFIEFPMFGKTFRRRNILQDANKTYLQTCHY
HPERDPFCPVFRIDDIVSFAGENFTQLAVRGGVIVISIEWNCNLDLDFMELCKPIYTFRRVDDPNT
NIAPGWNFRYANYHEENRRTLIKAYGIRFVIEVRGQGGKFNILPTLLNIGSGLALLGVTTVMCDFI
ILYFTKNRTFYKGVKYLLVDGEDAEKEPLQSESGLTYGSDRNQ
>cbriggs_x
MDALVKSRLDAIFNGKKVPPLDFVEQFEFTANVKKEDGLGLELKHLMVVSITYNSPCQGQIKVG
DVLLSLNGTPITGQDKMGKLIQTIFNGQLTTKMSVKVMRLKRRIPRPSTFPPLYKHEGYTNDTLV
LYNLKGYFHLGLDIKELDGKLIVCDFVENSLADITFSLGEAILDVDGEKTSTCTSFNDRVRKSLEI
RNYCLVTVEVPSTDPLKNLLRNQITKAVKDVGRINKLPLDAATYAAEGVAIFKKCCDGPLKSAY
VGDKHGNRTSENASRLKMEDKVKETDVPTGWNSRLFVRLPPAKTLESETLSQ
>trich_x1

Journal of Experimental Biology • Supplementary information

MENCCNRFADLALDYNTPKIIHIKSKVVGFINRFIQLAIVGYIIGYVLVYKKGYQEFDTAQNSVTS
KVKGVAFVNYTKDPNIGTRVWDPADYIIPPEENGAFFVMTNMIITKNQTNTVCPEDMSIRGANCT
DSTDCIPGKHLYLGHGVNTGECVPVSPDSDEMTCEIYSWCPLEYDHLRTNYPFLGEAVNFTVLIK
NSIAFPKFNVRRSNIDAVKNSEDLKHCMYDPVHDALCPILKLGTIVNSAEQDFNKIAYKVNNKLH
RDLIKAYGIRFVFIIVGRAGRFSVVPLLLNIGSGLALLAIASVISDVVILYVLKRRQYYRSKKYQTV
SPFDDDSPLLQEDVE
>salpin_x
MAGGEGKFSCTQAALSVLEYDTLKTVHIKSKKVGLIFRILQLVILGYIIGYAIIWQKGYQAADQA
VSTVYSKVKGVAVTCNDLNVTSIRNCSDSDIRVWDTADYVIPPQESNAAFIVTNSVQTSNQTQQP
GGWPEDPDASTKGSQRAYTCEKNADCEAVRFSPSRNGAVSGKCDLATKRCMVYGWGPVELSK
DDDASQVLGYPRQMPQVKNFTMFIKNTIFFPHFQRKFGNTGLGNSSASYLKSCLWNESTDRYCP
VFRIEDILSAAGVKSFEQDVMMAGAVITVQIRYDCNLDKNVETCAPEYRFTRIDEPNNTLSNGFN
FRFARYAIGEVPSRDLYKVYGLRFVFIVSGTAGKFDFVPLLVTFGSGLGLLSLATIVADLLVTKCL
RNAEFYYDRKYEIVNEDEVEGEAINVTIRQKPEEDTPLLH
>pleuro_x
MASGLGSLDDTSDSPNGNGKSFRRKGCLLIVTLFYENTLSTWWGTTPLRYRYTVERVPYTPYRV
EEVIPMVTPSLLFSSTVPLNTATAHDRYTNVQHRLLRKRYGVHLKFVQGGSLGVFSITCLLQCLA
TSMSLITLITTVGDIIALYLLPQSGFYR
>aplysia_4.1a
MAPPQVMKSAARSALGVFFEYDTPRIVHIRSKKVGLFNRFLQLSILAYIIGYAIVYKKGYQEFENV
QSAVTTKVKGIAFSNGSIPGIGTRTWDVADYVIPPQENDAFFVMTNVVVTPGQTQTGCAEDPGV
TGAICQTNADCEQVKGTILPSGSGPVTGECVPSDVPGSKEKVCQIFGWCPLENDKMQGDSPVLE
NAKNFTVFIKNNIEFPRFGVSRRNILGFYNDTALKTCRWKRGDPKLKFCPIFVLDDIARDAGVTFE
NMMMEGGVMQIVIDWTCNLDYSVEDCVPEYTFRRLDKGDYSVSRGFNFRFADRYSVFNESTGL
QLYRNLYKAYGVRFLVTVQGKAGKFSIVPLLLNIGSGMALLGVATIICDIMVLYVLKAKNFYRD
KKYLDVKGQDAFEVLEEEAGPGEQMSEGSTNSVSRRNLTASDGGAAP
>aplysia_4.2
MAPPQVMKSAARSALDVFFEYDTPRIVHIRSKKVGLFNRFLQLSILAYIIGYAIVYKKGYQEFENV
QSAVTTKVKGIAFSNGSIPGIGTRTWDVADYVIPPQENDAFFVMTNVVVTPGQTQTGCAEDPGV
TGAICQTNADCEQVKGTILPSGSGPVTGECVPSDVPGSKEKVCQIFGWCPLENDKMQGDSPVLE
NAKNFTVFIKNNIEFPRFGVSRRNILGFYNDTALKTCRWKRGDPKLKFCPIFVLDDIARDAGVTFE
NMMMEGGVMQIVIDWTCNLDYSVEDCVPEYTFRRLDKGDYSVSRGFNFRFADRYSVFNESTGL
QLYRNLYKAYGVRFLVTVQGKAGKFSIVPLLLNIGSGMALLGVATIICDIMVLYVLKAKNFYRD
KKYLDVKGQDAFEVLEEEAGPGEQMSEGSTNSVSRRNLTASDGGAAP
>Em0003g54a
MLNYVTYKYVVLKDFRLGILYYVLVASIVLYTLTEIFFNKGYLHFDTRPQGTIKIVISDNLSNETF
RPASLPYCQGNAACKFIDPMDLSWPVESRAITIATYIKDRDQLLEEDTNSPEDEYVLKREQDYFT
QGAEYIVMKVDHAVVAHDSSGRERLAASQRKLKGYLVDLNNRPVVYLSPGEGKPDKMSLQQL
LQAAGVHSLDEQSDAVSARGRSFRERGCVLRVTIFYENWYSTWFGTSVREVQSPGHRDSTRREE
GQERLFRKRYAVRVEFIQAGSIGLGTSTLVAASSQHHLWTCSPQPPSSSMLTTATQLLHAHHSHP
APPCSPQPPSSSMLTTATQLLHAHHSHPAPPCSPQPPSSSMLTQPPAPHAPHSPSSSMLTTATQLLH
AHHSHPAPPCSPQPPSSSMLTTATQLLHAHHSHPAPPCSPQPPSSSMLTTATQLLHAHHSHPAPPC
SPQPPSSSMLTTATQLLHAYHSHPAPPCSPPSSSMLTTQLLHAAPPCSSSMQLLHAAPPCSSSMQL
LHDEVLKGVWLVLHPIGSGDIWQLGQRGHDTISRPASHLLAIHQSSPKSSVVAVSYGGTSKRPHT
LPIDIALASVPRPYITAPDVHVLLHRYKNWNDLKPKIDLEVIKRDQKMKQTMKSLKVELNENYV
GLKIDLEVIKRDQKMQQTMKSSKVELNKLQILVYQSLVTEQVTDDTEVMMAELKVTIQIADLKL
VIERLGLIRDFLMVVACEEMSEEKQCFRHHL
>Em0004g666a
MISEILKSTRSDYLRYSIPKYIVFKGNCIALFYTLSGLILVLYTVLYGIIYAKSYLYFDNHPQGTMKI
VLSDARSVDWNLQLENLTYCHGNISCKDADPMSLNGPVETRAVTIATFIKERKQMLTNSTNSDH
YGTYSEEQYYTKGPEYVVLKIDHAITASDSSGKIFLRASHRKLTGYLMDINGNPVRQLNSDQRSG

Journal of Experimental Biology • Supplementary information

KPDKLSLIELLKAAGVESLEKPSDSISSNGRSFRKRGCVLQVNIFYHNWLSTWFHTSNAQYEYRV
HRVPYIDYSTKEIINSSVQINPLERLLRKRYALRIEIVQSGSLGIFSLSALLSTIVNGNIVFTIAIWIIEK
IAISRYQSYIEEEYDEANYYHARTHLCAKIRPVKIYLFIFCIAIVAFSFFIAQYGN
>Em0004g75a
MNLCRSAWGTLASFAFDYDTPKIVRIQNKKVGALYRLFQLGIMGFMIGYSIIYSKGYQDKDNVF
GGVTMKLKGVEFYNSSQEPFLQNCQGSTGAQVYDPADYVVPPQEPNSFFVMTNMWVTCDQSY
GFCGEDPSVQGSNCTGPSDCVKQNTIKGNGPRTGVCNNETHTCEVYAWCPTEKENVSYTDMGP
KINAEGFTVLIKNSIQFPNLAPNTLRKNYISNYDLSKCGIWSHTTITSSDLPAGDIVAMIDPPQNFTE
LALREQIGVVLDDCFGTCDLDSVWL
>Em0004g76a
MRPKEKCEWAWSVFIAATFEYDTPKIVHIRNKKVGVLYRLFQLCIMGFMIGYSIIYSKGYQDTQT
VIGAVTTKLKGVQYYNVTGGVPFLQNCVATNEPLVLDPADYVIPPEQPSSFFVMTNMWVTCNQ
TYGTCVEQDPSLASKCSGPADCTPQSTIKGDGPRTGTCNNQTHTCDVYAWCPTEYENTNFTNMG
PKINAESFTVLIKNTIQFPNLDPNFFKKNYISNGDLANCTNSLIGNNYCPIFKLADIVNMISEEPKNF
SNLATKGAVVVLTITWNCDLDMSLDHCNPRYSARRLDTTPLDTASNSGGFNFRFAKYWYDSNG
VQYRTLIKAYGISFVVELVGQGGKFSFTALIFKLGSTIALLGIAAVLSDMVVLYVVQKRHLYHQA
KYQVVNDEDDSVAGDDSHYHSVHPLLNK
>Em0004g77a
MGPKEKCEWAWSVFVAATFEYDTPKIVHIQNKKVGVLYRLFQLCIMGFMIGYSIIYSKGYQDTQ
TVIGAVTTKLKGVQYYNATGGVPFLQNCVATNGPLVLDPADYVIPPEQPNSFFVMTNMWVTCN
QTYGTCVEQDPSLASKCSGPADCTPQSTIKGDGPRTGTCNNQTHTCDVYAWCPTEYENTNFTNM
GPKINAENFTVLIKNTIQFPNLDSNFFKKNYISNGDLANCTNSLIGNNYCPIFKLADIVNMISEEPK
NFSNLATKGAVVVLTITWNCDLDMSLDHCNPRYSARRLDTTPLDTASNSGGFNFRFAKYWYDS
NGVQYRTLIKAYGISFVVELVGQGGKFSFTALIFKLGSTIALLGIAAVLSDMVVLYVVQKRHLYY
QEKYQVVNDEDDSNHSEEGPLLNK
>Em0004g80a
MTNMWVTCNQTYGTCVEQDPSLASKCSGPADCTPQSTIKGDGVLCLGTGMLDVWSLRCNMGP
KINAENFTVLIKNTIQFPNLDSNFFKKNYISNGDLANCTNSLIGNNYCPIFKLADIVNMISQEPKNF
SNLATKGAVVVLMITWNCDLDMSLDHCNPRYSARSHMGRINHYISNLFK
>Em0002g1083a
MWLTCNQSVGICGEDERSVPNGQAYCLNNTNCTDGKLLRNGNGPQTGTCNLQTKTCNVLAWC
PVEAERKESNLKPKIDAANFTVLVKNTVSFPNSVSNYARSNIYGGDEHWLKNCTYDKDLSPGCPI
FTLGQIVSMISPTENFTELSITGAVIAFNIEWNCDLDETWLQPTCTPVYSVAREDRQNGVSAGYHF
RSTRNRYETNGTQYRTLVKAYGIRVIVRVNGLGGKASLIAVARWLGTSLAYFTSATGFLTLLIVY
GYLNYYIQVQELIKSSKIYNCCGCKGKIGNSILYGLISGFCCCCILLPIICDCGKDKSESNNPEDEKK
DENKDANKDYPTERILKLLAGAINVIWILVLVIFMVIPVLTMFMIVVLASILSLVVLCCLRYYKVN
TTKRLTKVLNTVLSYEIHDTHAAAILKRKEGVQKYRDAKEKEKGCAPNHVVITFDDAPDEN
>Em0001g2994a
MEEGEALDSFGLAPYLFEPTGHGLELSTDDDSDDGDEPPVLPAVRLGNTSWCFCGKCSALATKP
ECMCCKELPDTRHLFDEEGMSCISEHPEFAAACLQPLVLRVALVGMMRIRGDPIAFPIPNR
>Em0001g3566a
MRGEKLVKANELLVKALTANLLPPALMDSAEIQEIASVVKDLVNVEFMLENRLDSISTDQGANFI
AAVQLLIEEGASEEQVRCACHKLQLSIKNSLEEKSTPSAYALVCLFRTITTTINNSPLLLDALKKR
QHSPEQLIIDSDKSGDEECSDNEEEIDPTNVLSGRKHGLKLIKDAVPFGEATEAMQERWRALDVS
FGKRRTEMAASFERETAEKANEERPMRQQRKDNLGSRMRRALAALQRQKQFLVFQKADKGSVI
VVEKGEDYIRNEREHLAVPQVYQRLHRDGRELAQAIKQAVYLRLRRLHNAGRLTEEEYKYCIPP
PKVPVLADAVEVLPGKDQKQHSTPQQHPTFLQEQVHDQVYDDDVPDDDSQQMQDSQYDDDDS
QYDDDVYDDVQDQGQDDDDVQDQGQDSQYDDDDEACTKEMAAAIERMKELKIYDSTRKVQL
KWIMII
>Aqu003385281

Journal of Experimental Biology • Supplementary information

MLNYVTYKYVVLKDYRLGLFYYILAGLIILYTLVEILYNKGYLEIDSKPVGFVRAVVSDDLPLLN
ASSLSYCTSNHSITNNISCYYETPHELNWPVESRSLSIMTFAKDKLQASLSLSPDSDEFEGINETQY
FTLGPEHVLVKVDHAVVASRFGSGRDQLAASKRQMIGYLLDSRGALIRKLSIPGKPDKITLQELL
EAGGVSGLDEPSDALNAKGQSIRQRGVVIIVSIYYQNWFNTWFGTSDIEYSYQVRHIPYMDYNSK
QLLPAMPPHSDDGTGRKWQLLRKRYSVRVEFQQTGSLGMFSFSSLLLKLVSGVGLLTLTATIIDT
VALYLLPNRMLYRKHVYDESGDIKKKN
>Aqu019850128
MLNYVTYKYVVLKDYRLGLFYYILAGLIILYTLVEILYNKGYLEIDSKPVGFVRAVVSDDLPLLN
ASSLSYCTSNHSITNNISCYYETPHELNWPVESRSLSIMTFAKDKLQASLSLSPDSDEFEGINETQY
FTLGPEHVLVKVDHAVVASRFGSGRDQLAASKRQMIGYLLDSRGALIRKLSIPGKPDKITLQELL
EAGGVSGLDEPSDALNAKGQSIRQRGVVIIVSIYYQNWFNTWFGTSDIEYSYQVRHIPYMDYNSK
QLLPAMPPHSDDGTGRKWQLLRKRYSVRVEFQQTGSLGMFSFSSLLL
>Aqu003384136
MSAASACRWLGSTTVSVLFEYDTPKFVHIRNKKVGLLNRLVQLAIVGYIIGYGIVWENGAQDKE
PVQSVVTTKVKGVSVLNATRQFMTDCNEWPILDHVDLIVPAQEPNSFFVTTNLWATCNQKYGV
CPELSTIPDAVCLTNDSCPPGIIYRTGNGVTTGECTGFNETCQIRGWCPVEDENDTLTDEGPIIDTK
DFTVLVKNSISFPKLDSSYRVRNIPERANNKSYLSGCQFDPDSEMGLFCPIFSLKQIVDMINSSDGY
YESLATKGAVVGLEISWDCNLDRSPTHCVPKYSARRLDNPNAQISNGFNFRYPRYYRDQNGDDV
RDVWKVYGIKFEIVVTGEGRKFSFTTLVLALGSTIALLAIATTVTDVIALYLLKKGTYYREKKYQ
KVAKEDEESVTLKALNHSQEKSPLLREKRK
E
>Aqu019863983
MAAHATRSSAVEDESNHGEGQIFCSCKGTCSRKKGKGFCPCKAAGVNCNNSCLCGRKGPCKNK
LQVTSAATGVTGHAVDQDDAIDEQIQQMDDFIDGLSSQDSKKLIKELLLGRGGVALAQSLLDRE
PSDPHEPPHPHYIQPPWCTCGKCTIMDSEEEHVCCKRQTCITVYRHFFNICLDQQVLTVAIHQRSD
IRAEDISYSPESFRKAAYRQYILWKYKKLGKGNRRVCPSCVVNCIREWYPSSTGRYMGFRSE
>Aqu011403990
MSDSEGTNEWSTDSENEAEESLGTQPFMFEPEASDSSSSDSDDEINDPESRLGNTTWCQCGHCVP
MPTCMECVCCCEIQQVVAKKNHLQSPVVCITLHPGFHNVCLDMWVLQAASYVYRQQYGTSAR
HGSFHDQMRHTAYRQLVSWCWQWLGRNNRVVLPACAVAKIRETFPSNGNYVGFEL
>Aqu003388934
MASCTSDSDEEPVDYSNSLDILPYNFEPEGSSEEESASESDSSHDDDELSSTTWCSCSNCVIMPSRR
ECLCCRDIQAIMSKINEVNDTQVKCITEHPGFSAVCLNVWVLQTAYAQYRQQYGNYNASMHER
HRYTAYRQLVRWCWGLLGREVRVILPSCAVSIIRTTFPSSAYTGYSNP
>Spo9476
VHADFNRPTPISQRTYKKYVVLKDARLGILYYVLVGSILLYTLVEIFVQKGYLHFDTHPQ
GTIKIVISDDLSNETFRPASLPYCQGNVKCKYVDPIDLNWPVESRAVTIATFVKDRDQLL
EEDTNSPDDEYILKREQDYFTQGPEYVVLKVDHAVVASDPSGRERLAASQRKLKGFLVDL
DNKPISHLSPGEGKPDKVSLQQLLTAAGVESLDEISDAVSARGRSFRERGCVLRVTIFYE
NWYCTWFGTSDIQYTYHVRRIPYMDYSVREVQSPHKDGTLQSSREQGQQRLFRKRYAVRL
EFIQAGSLGMFSLTTLLLKLVSGVGLL
>Efr15342
LKDARLGILYYALVSAILLYTLVEIFVQKGYLHFDTHPQGTIKIVISDDLSNETFRPESL
PYCQRNISCKYVDPMDLNWPVESRAITIATYVKDRDQLLEADTNSPGNEYVVKREQEYFT
QGAEYVIMKIDHAVVAKDASGRESVAASQRKLEGYLVDLDNRPIAHLSPGEGKPDKVSLQ
QLLSAAGVVSLDESSDAVSARGRSIRER
>Spo22477
LSTKSYLYFDTHPQGMMKIGVSDDMSCNNSIHSDNLSYCLSNITCKCADPMFLNWPVESQ
AVTIATFIKERKQLLNNTNGQNIYDTFSEEQYYTKHPEYVVVRIDHAITASSSSGSVFLT
ASHRQLKGYLTDQNGNHIKLFSGEGKGKPDKVSLADLLKAAGVENLEMPSDAINSKGKSF
RKRGCILQVKIVYNNWHYTWFSTSDIRYEYQVRRIPYMDYSKKEIEPPLQINSSHRMLRK

Journal of Experimental Biology • Supplementary information

RYSLRVVFVQSGSIGIWL
>Efr14663_5
SCAHMAVNLCRSAWSALFSLGFDYDTPKIVRIKNKKVGVLYRLFQLGIMGFMIGYSIIYS
KGYQDKDGVMGGVTMKLKGVEFYNASSEPFMQNCAASTGGQVFDPADYVIPPQEPNSFFV
MTNMWITCDQSYGTCGEDPSVKESSCTGPSDCVPQNTTKGNGPRTGRCDNSTNTCEVYAW
CPTEKENANYTATGPKINARGFTVLIKNSVQFPNLAPNRLRKNYISNNDLSSCGIWSRDN
YYCPIFVLGDIVAMVNPPQNFTQLATEGAVIVLTITWNCDLDRTNCDPQYDARRLDATAY
EEEGAGGFNFRFAKYWYDNQGTQYRTLVKAYGILFIVELAGQGGKFSLAALFLKLGSTVA
LLGIAAVLSDMVVLYVVQKRHLYWEQKYLMVNEDATATEKEGGTEREKEQLLK*
>Efr14663_4
SCAHMAVNLCRSAWSALFSLGFDYDTPKIVRIKNKKVGVLYRLFQLGIMGFMIGYSIIYS
KGYQDKDGVMGGVTMKLKGVEFYNASSEPFMQNCAASTGGQVFDPADYVIPPQEPNSFFV
MTNMWITCDQSYGTCGEDPSVKESSCTGPSDCVPQNTTKGNGPRTGRCDNSTNTCEVYAW
CPTEKENANYTATGPKINARGFTVLIKNSVQFPNLAPNRLRKNYISNNDLSSCGIWSRDN
YYCPIFVLGDIVAMVNPPQNFTQLATEGAVIVLTITWNCDLDRTNCDPQYDARRLDATAY
EEEGAGGFNFRFAKYWYDNQGTQYRTLVKAYGILFIVELAGQGGKFSFATLVLKLGSTVA
LLGIAAVLSDMVVLYLLRNRHLYYEQKYLMVEERERDAEHGGSINFDEKKPLLARENI*
>Efr14662_2
SCAHMAVNLCRSAWSALFSLGFDYDTPKIVRIKNKKVGVLYRLFQLGIMGFMIGYSIIYS
KGYQDKDGVMGGVTMKLKGVEFYNASSEPFMQNCAASTGGQVFDPADYVIPPQEPNSFFV
MTNMWITCDQSYGTCGEDPSVKESSCTGPSDCVPQNTTKGNGPRTGRCDNSTNTCEVYAW
CPTEKENANYTATGPKINARGFTVLIKNSVQFPNLAPNRLRKNYISNNDLSSCGIWSRDN
YYCPIFVLGDIVAMVNPPQNFTQLATEGAVIVLTITWNCDLDRTNCDPQYDARRLDATAY
EEEGAGGFNFRFAKYWYDNQGTQYRTLVKAYGILFIVELAGQGGKFSFATLVLKLGSTVA
LLGIAAVLSDMVVLYLLRNRHLYYEQKYLMVEERERDAEHGGSINFDEKKPLLARENI*
>Efr14663_1
SCAHMAVNLCRSAWSALFSLGFDYDTPKIVRIKNKKVGVLYRLFQLGIMGFMIGYSIIYS
KGYQDKDGVMGGVTMKLKGVEFYNASSEPFMQNCAASTGGQVFDPADYVIPPQEPNSFFV
MTNMWITCDQSYGTCGEDPSVKESSCTGPSDCVPQNTTKGNGPRTGRCDNSTNTCEVYAW
CPTEKENANYTATGPKINARGFTVLIKNSVQFPNLAPNRLRKNYISNNDLSSCGIWSRDN
YYCPIFVLGDIVAMVNPPQNFTQLATEGAVIVLTITWNCDLDRTNCDPQYDARRLDATAY
EEEGAGGFNFRFAKYWYDNQGTQYRTLVKAYGILFIVELAGQAAVLSDMVVLYLLRNRHL
YYEQKYLMVEERERDAEHGGSINFDEKKPLLARENI*
>Efr14663_2
GGAIMAKCQWVWSVFSSAVFEYDTPKIVHIRNKKVGVLYRLFQLGIMGFMIGYSIIYSKG
YQETQTVIGAVTSKLKGVQYYNATGEEPFMQNCVGASGPLVFDPADYVIPPQEPNSFFVM
TNMWITCNQSYGTCLEQDPTLASKCSGPFDCGPQSTIKGDGPRTGACDNDTRTCEVFAWC
PTEYENTGFTNLGPKINAGNFTVLIKNSIQFPNLDPDFFKKNYISNDDLANCTTSSHANS
YCPIFKLADIVNMVSQAPKNYDTLATKGAVVVMTITWNCDLDFPPDHCNPTYSARRLDTT
SKDTANNTGGFNFRFAKYWYDNQGTQYRTLVKAYGILFIVELAGQGGKFSFATLVLKLGS
TVALLGIAAVLSDMVVLYLLRNRHLYYEQKYLMVEERERDAEHGGSINFDEKKPLLAREN
I*
>Efr14662_1
AIMAKCQWVWSVFSSAVFEYDTPKIVHIRNKKVGVLYRLFQLGIMGFMIGYSIIYSKGYQ
ETQTVIGAVTSKLKGVQYYNATGEEPFMQNCVGASGPLVFDPADYVIPPQEPNSFFVMTN
MWITCNQSYGTCLEQDPTLASKCSGPFDCGPQSTIKGDGPRTGACDNDTRTCEVFAWCPT
EYENTGFTNLGPKINAGNFTVLIKNSIQFPNLDPDFFKKNYISNDDLANCTTSSHANSYC
PIFKLADIVNMVSQAPKNYDTLATKGAVVVMTITWNCDLDFPPDHCNPTYSARRLDTTSK
DTANNTGGFNFRFAKYWYDNQGTQYRTLVKAYGILFIVELAGQGGKFSLAALFLKLGSTV
ALLGIAAVLSDMVVLYVVQKRHLYWEQKYLMVNEDATATEKEGGTEREKEQLLK*
>Efr14663_6

Journal of Experimental Biology • Supplementary information

GGAIMAKCQWVWSVFSSAVFEYDTPKIVHIRNKKVGVLYRLFQLGIMGFMIGYSIIYSKG
YQETQTVIGAVTSKLKGVQYYNATGEEPFMQNCVGASGPLVFDPADYVIPPQEPNSFFVM
TNMWITCNQSYGTCLEQDPTLASKCSGPFDCGPQSTIKGDGPRTGACDNDTRTCEVFAWC
PTEYENTGFTNLGPKINAGNFTVLIKNSIQFPNLDPDFFKKNYISNDDLANCTTSSHANS
YCPIFKLADIVNMVSQAPKNYDTLATKGAVVVMTITWNCDLDFPPDHCNPTYSARRLDTT
SKDTANNTGGFNFRFAKYWYDNQGTQYRTLVKAYGILFIVELAGQGGKFSLAALFLKLGS
TVALLGIAAVLSDMVVLYVVQKRHLYWEQKYLMVNEDATATEKEGGTEREKEQLLK*
>Efr14662_3
AIMAKCQWVWSVFSSAVFEYDTPKIVHIRNKKVGVLYRLFQLGIMGFMIGYSIIYSKGYQ
ETQTVIGAVTSKLKGVQYYNATGEEPFMQNCVGASGPLVFDPADYVIPPQEPNSFFVMTN
MWITCNQSYGTCLEQDPTLASKCSGPFDCGPQSTIKGDGPRTGACDNDTRTCEVFAWCPT
EYENTGFTNLGPKINAGNFTVLIKNSIQFPNLDPDFFKKNYISNDDLANCTTSSHANSYC
PIFKLADIVNMVSQAPKNYDTLATKGAVVVMTITWNCDLDFPPDHCNPTYSARRLDTTSK
DTANNTGGFNFRFAKYWYDNQGTQYRTLVKAYGILFIVELAGQAAVLSDMVVLYLLRNRH
LYYEQKYLMVEERERDAEHGGSINFDEKKPLLARENI*
>Efr14663_3
GGAIMAKCQWVWSVFSSAVFEYDTPKIVHIRNKKVGVLYRLFQLGIMGFMIGYSIIYSKG
YQETQTVIGAVTSKLKGVQYYNATGEEPFMQNCVGASGPLVFDPADYVIPPQEPNSFFVM
TNMWITCNQSYGTCLEQDPTLASKCSGPFDCGPQSTIKGDGPRTGACDNDTRTCEVFAWC
PTEYENTGFTNLGPKINAGNFTVLIKNSIQFPNLDPDFFKKNYISNDDLANCTTSSHANS
YCPIFKLADIVNMVSQAPKNYDTLATKGAVVVMTITWNCDLDFPPDHCNPTYSARRLDTT
SKDTANNTGGFNFRFAKYWYDNQGTQYRTLVKAYGILFIVELAGQAAVLSDMVVLYLLRNRHL
YYEQKYLMVEERERDAEHGGSINFDEKKPLLARENI*
>Efr16401_12
MDIQAPYNSMDYDSFFVVTNLWITCDQSVGICGEDLNSAKESGCVNDTDCSNGKVLLSGNGPQT
GTCNITTKTCNVLAWCPVEAESNESTQYPKIDASNFTVLIKNSVSFPTSSSKYTRSNIYGGYEQSG
LYNCIYDKDKSPGCPIFTLGQIVEMISPKQNFTELSRKGAVIIFNIEWNC
ILDEPWTHHDCIPVYSVTRQGGDSSSFDGYNTRFTTNRYVTNGTLYRTLVKAYGIRVIVR
NNGQGGKASINAVTTWLTKSLSYFSAVTTILIVLLVYGYLTYYIEVINYKKEDKKKSCCN
LLYILKGIIYFLLSGICCSCIILPVIYRTVIEKCKCNESKTKKIQKCIDYFTIDCNLRFI
ANVINICWVICIAILIGIPLIVLALIMFLIFVIPSGVAFLLSKVCDKKDRLTEVVNKVLR
YELHYNTYAAQILRKTWDNIEKFQEEKTEEQTGSKDTAKHVVIEFASCTAP*
>Efr16613_9
MDIQAPYNSMDYDSFFVVTNLWITCDQSVGICGEDLNSAKESGCVNDTDCSNGKVLLSGNGPQT
GTCNITTKTCNVLAWCPVEAESNESTQYPKIDASNFTVLIKNSVSFPTSSSKYTRSNIYGGYEQSG
LYNCIYDKDKSPGCPIFTLGQIVEMISPKQNFTELSRKGAVIIFNIEWNC
ILDEPWTHHDCIPVYSVTRQGGDSSSFDGYNTRFTTNRYVTNGTLYRTLVKAYGIRVIVR
NNGQGGKASINAVTTWLTKSLSYFSAVTTILIVLLVYGYLTYYIEVINYKKEDKKKSCCN
LLYILKGIIYFLLSGICCSCIILPVIYRTVIEKCKCNESKTKKIQKCIDYFTIDCNLRFI
ANVINICWVICIAILIGIPLIVLALIMFLIFVIPSGVAFLLSKVCDKKDRLTEVVNKVLR
YELHYNTYAAQILRKTWDNIDKFQEEKTEEQTGSKDTAKHVVIEFASCTAP*
>Syc3454
LGNTSWCECGKCVAMPDRMDCLCCHDAAVTLERSRESGAPCVTAIADFDTVVLHRAVLATALR
GYFERRKQPMPVSEQPDNRYVCCTVFVRQAYPCRVY
>Spo10686
CAIILTYVQEPHRKLPHCLKVLLTVFFLKPKTGISEAETDKLNDLLILSFVSAILPTSLV
DNAHFREFIHSLNDSYSLPHRTKLTKMIDSKFAEVKQKLQSLLEEASSVAITTDIAVIKH
TGDSYVTVTGHWVTKEWKLMAAVLGVYICDKSHTGEEIFDLVQYVKKDFVLADRLDAITTDNG
ANFVKAVRLMIEHGVAEEDIRCACHTLQLSIKKSLESPRQSHHLVVMFRTIVNLIMDSPILLKALR
DKQNDPSLHFNFEFESTVEDIETDNDQFITEYLHWGNTLRML*
>Aphr10395

Journal of Experimental Biology • Supplementary information

NKTKYSMSSFLDYTTYKYVTVRDKRLGVTYYIFVFLIFAYLIQDIFVDKGYLKIDTNPHG
TLRILVKDPNNEAPITALPYCPGFNTDLPTKFQYNRTDPCIDYDAQELSWPVEAHAVNIM
TFGKDNWQERKKSEKGTYNFETITEKQYFTRNPESTILKIDHSILSTQFSGKGFLGGSHR
TMVGYLEDKNGKIIRQISDPEKPDKLKVEELLMAAGINTLDQDSDALSGQGESYRMRGLI
LKVSIYYTNHEHMWFGTGQIRYTYRIQHVPLVDYQTKQLIPIMKDLPDEQIESATFHTNK
RLLRKRYSLRMIFEQEGSLGKTSLNALVYQLIAGTSLLTLLATVIDVVALNLIPTYSRYM
MDSSPELGKKEEKKDKKSSEKDIVSVDENVGGEKESKKDQ*
>Aphr14669
NKTKYSMSSFLDYTTYKYVTVRDKRLGVTYYIFVFLIFAYLIQDIFVDKGYLKIDTNPHG
TLRILVKDPNNEAPITALPYCPGFNTDLPTKFQYNRTDPCIDYDAQELSWPVEAHAVNIM
TFGKDNWQERKKSEKGTYNFETITEKQYFTRNPESTILKIDHSILSTQFSGKGFLGGSHR
TMVGYLEDKNGKIIRQISDPEKPDKLKVEELLMAAGINTLDQDSDALSGQGESYRMRGLI
LKVSIYYTNHEHMWFGTGQIRYTYRIQHVPLVDYQTKQLIPIMKDLPDEQIESATFHTNK
RLLRKRYSLRMIFEQEGSLGKTSLNALVYQLIAGTSLLTLLATVIDVVALNLIPTYSRYM
MDSSPELGKKEEKKDKKSSEKDIVSVDENVGGEKESKKDQ*
>Spo14633
GGPRPTSTMSDNSSDSGDLSLSCSFSPVESDQSDVSEVEDGLQTIEPYQFEPVASESDSH
TEPEAVVVDDGDPGDEERLHSRNWCQCGQCVIMPTARECVCCCEIEQVMSKKEESARRIT
CITEHEGFDSVCLNVWVLQAAYFSYRYHYGNTEEREIHEQYRFIAYRQLVGWCWGWLGRHVR
VVLPSCVVNKIKDYISLYYLCWI*
>Syc21026
CWVKHATSPDFATAAGLSRQHRQSTPRSISLQFVHPRQWTFPARPPRPSSSSTSELSVRA
AGASSSSGVMHGHNIENITSEYLIVFSVTLSRTVGVHILDGCNTIPRIGCDRLLSSRLLM
LPGYVAVG*

Fig. S1. Supplemental PDF file showing P2X sequences from all organisms examined

Journal of Experimental Biology • Supplementary information

```
                          1 .........10........20........30........40........50........60
mmus_1                    1 FFEYDTPRMVLVRNKKVGVIRLIQLVVLVYV-IGVFVYEKGYQTSSG-LISSVSVKLKGL
human_1                   1 LFEYDTPRMVLVRNKKVGVIRLIQLVVLVYV-IGVFLYEKGYQTSSG-LISSVSVKLKGL
mmus_4a                   1 LFEYDTPRIVLIRSRKVGLMRVVQLLILAYV-IGVFVWEKGYQETDS-VVSSVTTKAKGV
human_4.1                 1 LFEYDTPRIVLIRSRKVGLMRAVQLLILAYV-IGVFVWEKGYQETDS-VVSSVTTKVKGV
ggal_4a                   1 LFEYDTPRIVLIRSRKVGLIRAVQLAILAYV-IGVFLWEKGYQETDS-VVSSVTTKVKGV
xlae_4                    1 LFDYDTPRIALIKSRKIGLLRFIQLGILAYV-IGVFIWEKGYQEFDI-VVSSVTSKVKGV
drer_4b                   1 LFDHGTPVILVVSSKKVGFIRLLQLCIIVYV-AVVCWYKRAYQERDS-VISSVSTKVKGN
hvul_4a                   1 LFHYETVKIVDIKNKKVGALRFIQLIILAYV-IGVIVYKKGYQDFDN-AISTVTTKLKGT
nvec_4a                   1 LFEYDTEKIVHIKNKKIGIIRVIQLVIIAYI-IGVIVYKKGYQEFES-PYSSVTTKLKGV
amil_4a                   1 FFEYDTNKIVHIKSKKVGLIRFIQLVIIVYI-IGVIVYKKGYQEFQE-PYSSVTTKVKGV
tadh_x1                   1 ALDYNTPKIIHIKSKVVGFIRFIQLAIVGYI-IGVLVYKKGYQEFDT-AQNSVTSKVKGV
mmus_5a                   1 LFDYKTAKFVVAKSKKVGLLRVLQLTILLYL-LIVFLIKKSYQDIDTSLQSAVVTKVKGV
human_5.a                 1 LFDYKTEKYVIAKNKKVGLLRLLQASILAYL-VVVFLIKKGYQDVDTSLQSAVITKVKGV
drer_5                    1 LLDYKTEKFVIAKNKKVGVLRLFQLTVIGYL-IGVFIWKKGYQETEEAIQSSVITKLKGV
mmus_6a                   1 FLDYKTEKYVLTRNCRVGVSRLLQLAVVVYV-IGALLAKKGYQERDLAPQTSVITKLKGV
human_6.1                 1 LLDYKTEKYVMTRNWRVGALRLLQFGIVVYV-VGALLAKKGYQERDLEPQFSIITKLKGV
xlae_6a                   1 LLDYKTEKYLLTKNRKVGVIRFLHLAILCYI-IGVFLAKKGYQDTDSDPHFSIITKLKGV
ggal_6a                   1 LLDYKTEKFALTRNRRVGLLRLLQLAVLGYV-LGVFVVRRGYQDTDAAPRVSVVTKLKGV
acal_4.2                  1 FFEYDTPRIVHIRSKKVGLFRFLQLSILAYI-IGAIVYKKGYQEFEN-VQSAVTTKVKGI
cgig_4                    1 FFEYDTPRIVHIQSKKVGVIRFIQFCIIGYV-IGAIIFKKGYQDFDN-VQSVVTTKVKGI
Em0004g77a                1 TFEYDTPKIVHIQNKKVGVLRLFQLCIMGFM-IGSIIYSKGYQDTQT-VIGAVTTKLKGV
Efra14662_1               1 VFEYDTPKIVHIRNKKVGVLRLFQLGIMGFM-IGSIIYSKGYQETQT-VIGAVTSKLKGV
Efra14663_5               1 GFDYDTPKIVRIKNKKVGVLRLFQLGIMGFM-IGSIIYSKGYQDKDG-VMGGVTMKLKGV
Aqu003384136              1 LFEYDTPKFVHIRNKKVGLLRLVQLAIVGYI-IGGIVWENGAQDKEP-VQSVVTTKVKGV
mmus_3                    1 FFTYETTKSVVVKSWTIGIIRAVQLLIISYF-VGVFLHEKAYQVRDTAIESSVVTKVKGF
human_3                   1 FFTYETTKSVVVKSWTIGIIRVVQLLIISYF-VGVFLHEKAYQVRDTAIESSVVTKVKGS
ggal_3                    1 FFSYETTKSVVVKSWVVGVVRVVQLLILSYF-IGVFLHEKAYQVRDTVIESSVVTKVKGI
drer_3b                   1 FFTYETTKSVVVKSWTIGIIRVVQLIITYF-IGVFLYEKAYQVRDTAIESSVMTKVKGF
mmus_2d                   1 FWDYETPKVIVVRNRRLGFVRMVQLLILLYF-VWVFIVQKSYQDSETGPESSIITKVKGI
human_2.d                 1 LWDYETPKVIVVRNRRLGVLRAVQLLILLYF-VWVFIVQKSYQESETGPESSIITKVKGI
ggal_2a                   1 VSSYSSPQVVVVRDGRLGTARALQLLVLLYF-IGVFIVQKGYQERETGPESSVITKVKGV
mmus_7a                   1 VLQYETNKVTRIQSTNYGTVWVLHMIVFSYI--SALVSDKLYQRKEP-VISSVHTKVKGI
human_7                   1 VFQYETNKVTRIQSMNYGTIWFFHVIIFSYV--CALVSDKLYQRKEP-VISSVHTKVKGI
ggal_7                    1 VFNYESPKLIRFPSVGLVCVWFIYGVIAVYI-C-TLIVHKRYQEKEE-LTSSVRVTLKGV
drer_7                    1 LCEYDTQKLVKIKSVKLGSLWTLNGVILMFI-C-MMLWNKEYQEYDF-VVSSVTTKVKGV
spur_4                    1 --------MLAVGTNDVGLLESLSRGIATVF-DKVIFWQKGYQSTDN-VEGSATSKLKGV
sros_x                    1 VLEYDTLKTVHIKSKKVGLIRILQLVILGYI-IGAIIWQKGYQAADQ-AVSTVYSKVKGV
hvul_E                    1 LFHYITYKCIIIKDKRLGFAYSLALIILLYT-F-QIIINKAYLKFDYSPKGSIRLLVSTP
nvec_E1                   1 LLHYITYKLVVIRDKRLGTIYTFVAAIILFT-L-EIFVFKGYLEYDTSPEGTMRILVSDP
Aqu003385281              1 MLNYVTYKYVVLKDYRLGLFYIIAGLIILYT-L-EILYNKGYLEIDSKPVGFVRAVVSDD
Slac9476                  1 ISQRTYKKYVVLKDARLGILYVLVGSILLYT-L-EIFVQKGYLHFDTHPQGTIKIVISDD
Em0004g666a               1 YLRYSIPKYIVFKGNCIALFTLSGLILVLYTVL-GIIYAKSYLYFDNHPQGTMKIVLSDA
Avas14669                 1 FLDYTTYKYVTVRDKRLGVTYIFVFLIFAYL-I-DIFVDKGYLKIDTNPHGTIRILVKDP
Avas10395                 1 FLDYTTYKYVTVRDKRLGVTYIFVFLIFAYL-I-DIFVDKGYLKIDTNPHGTIRILVKDP
ddiscoideum               1 IFQYSTVKIVRIRDRRLGILLSFLVGIVAYI-VVSAIIKKGYLFTEV-PIGSVRTSLKGP
gmar_4_KAF0502320         1 IFGYQTFKMVRVKDLRLGVIRIFQLLILVYI-L-EIIYNQRYLKTETPVPGAIRVTLQAP
consensus                 1    y t k v vr  kvg i  i l il y  i   v  kgyq  d     sv  klkg
```

Journal of Experimental Biology • Supplementary information

```
                      61 ........70........80........90.......100.......110.......120
mmus_1                59 AVTQQVWDVADYVFPAHGDSSFVVMTNFIMTPQQAQGHCAENPCQDDSGCAQGIRTGNCT
human_1               59 AVTQQVWDVADYVFPAQGDNSFVVMTNFIVTPKQTQGYCAEHPCKEDSGCAQGIRTGKCT
mmus_4a               59 AVTNRIWDVADYVVPAQEENSLFIMTNMIVTVNQTQGTCPEIPCDSDANCSSGIGTGRCS
human_4.1             59 AVTNRIWDVADYVIPAQEENSLFVMTNVILTMNQTQGLCPEIPCKSDASCSNGVSTGRCS
ggal_4a               59 TMTNRIWDVADYVIPPQEKNAVFVMTNMIFTLNQSQSHCPELPCNNNSSCSNGIQTGACS
xlae_4                59 VVTNKIWDVADYIIPAQEENAVFVMTNLILTQNQTQGHCPELPCSKEQPCSNGVQTGKCT
drer_4b               59 ILTNHVWDTSEYVIPPQGENSFFVLTNAIVTQGQTQGSCPEIPCQSDSDCGNGVQTGKCT
hvul_4a               59 SYVNEVYDPADYVVPPQENNAFFVMTNMIITPNQTRSRCPEDPCQVDKDCGNGVRTGRCS
nvec_4a               59 ALTNFLWDSADLVIPPEEEGAVFMMTNMIITPEQSQGKCPEDPCTADADCSHGVNTGNCT
amil_4a               59 SLTNHVWDSSDFVIPPEEDNAVFVMTNMIISPNQTQSTCPEDPCTKDSDCGHGLRTGRCN
tadh_x1               59 AFVNRVWDPADYIIPPEENGAFFVMTNMIITKNQTNTVCPEDMCTDSTDCGHGVNTGECD
mmus_5a               60 AYTNRLWDVADFVIPSQGENVFFVVTNLIVTPNQRQGICAERECSEDTDCGHGLKTGRCT
human_5.a             60 AFTNRIWDVADYVIPAQGENVFFVVTNLIVTPNQRQNVCAENECSKDSDCGNGVKTGRCA
drer_5                60 DLTNQLWGAEDYVIPPQGDRVFFVVTNYLVTPNQRLGYCPESPCTNDNECGHGVKTGRCD
mmus_6a               60 SVTQRLWDVADFVKPSQGENVFFLVTNFLVTPAQVQGRCPEHPCWADEDCSHGIKTGQCT
human_6.1             60 SVTQRLWDVADFVKPPQGENVFFLVTNFLVTPAQVQGRCPEHPCWVDEDCSHGVKTGQCT
xlae_6a               60 SITKKIWDVADFVKPSQGENVLFLVTNFIATTGQVQRTCAENPCSENLDCGNGIKTGKCT
ggal_6a               60 SVSRRLWDAADFSRPPQGENVLFLVTNFIVTDRQVQGTCPESPCMEDVDCGNGIKTGRCT
acal_4.2              59 AFSNRTWDVADYVIPPQENDAFFVMTNVVVTPGQTQTGCAEDPCQTNADCGSGPVTGECK
cgig_4                59 VMVNPIWDVADYIVPPQENNAFFVITNLIVTPNQSIGICPEDSCTVDKDCGNGVLTGKCD
Em0004g77a            59 QYYNLVLDPADYVIPPEQPNSFFVMTNMWVTCNQTYGTCVEQDCSGPADCGDGPRTGTCQ
Efra14662_1           59 QYYNLVFDPADYVIPPQEPNSFFVMTNMWITCNQSYGTCLEQDCSGPFDCGDGPRTGACD
Efra14663_5           59 EFYNQVFDPADYVIPPQEPNSFFVMTNMWITCDQSYGTCGEDPCTGPSDCGNGPRTGRCS
Aqu003384136          59 SVLNPILDHVDLIVPAQEPNSFFVTTNLWATCNQKYGVCPELSCLTNDSCGNGVTTGEC-
mmus_3                60 GRY-RVMDVSDYVTPPQGTSVFVIITKMIVTENQMQGFCPENECVSDSQCGGGILTGRCV
human_3               60 GLY-RVMDVSDYVTPPQGTSVFVIITKMIVTENQMQGFCPESECVSDSQCGGGILTGRCV
ggal_3                60 GKY-RVLDTADYVTPPQGTSVFVVVTKQILTENQEQGVCPESECASNRDCGSGVLTGRCT
drer_3b               60 GEY-RIMDVADYVTPTQGASVFCVITKLITTENQVQGNCPESECENDIRCSNGLLTGRCT
mmus_2d               60 TMSEKVWDVEEYVKPPEGGSVVSIITRIEVTPSQTLGTCPESMCHLDDDCGNGIRTGRCD
human_2.d             60 TTSEKVWDVEEYVKPPEGGSVFSIITRVEATHSQTQGTCPESICLSDADCGNGLRTGRCP
ggal_2a               60 TQSLKVWDVGEYVAPPEGGSSFSILTRVEVSAAQAMGTCHEGPCHSEQDCHHGVRTGRCS
mmus_7a               58 AEVTSIFDTADYTFPLQG-NSFFVMTNYVKSEGQVQTLCPEYPCSSDRRCSKGIQTGRCT
human_7               58 AEVKSVFDTADYTFPLQG-NSFFVMTNFLKTEGQEQRLCPEYPCSSDRGCSKGIQTGRCN
ggal_7                58 ARVDRIWDAAEYTIPTQTRDSFFVMTNIIRTENQIQKTCPEYPCSSDKSCSNGVQTGKCT
drer_7                58 AKITVVWDVVDYSGPSQGKNSFFVATNAIVTKNQKQGNCAEILCRTDKDCSHGVQTGACL
spur_4                51 AYTNRVWDVSDYVIPAQQTNGFFVMTNMVLSPMQSRGACPEDPCTTDVECGNGIKTGRCA
sros_x                59 AVTCRVWDTADYVIPPQESNAAFIVTNSVQTSNQTQGGWPEDPCEKNADCRNGAVSGKCA
hvul_E                59 PFNNKRVDGLTLNWPVESKAVTIAT--FMKEIVQEKTNSINDI-----------------
nvec_E1               59 KDDNEYIDAQELSWPIESHTINLMT--FGKDRWQVKDSFLK-------------------
Aqu003385281          59 LP--YYETPHELNWPVESRSLSIMT--FAKDKLQASSLSPDS------------------
Slac9476              59 LSNEKYVDPIDLNWPVESRAVTIAT--FVKDRDQLLTNSPD-------------------
Em0004g666a           60 RSVDKDADPMSLNGPVETRAVTIAT--FIKERKQMLTNSTNS------------------
Avas14669             59 NNEAIDYDAQELSWPVEAHAVNIMT--FGKDNWQERKKSEKG------------------
Avas10395             59 NNEAIDYDAQELSWPVEAHAVNIMT--FGKDNWQERKKSEKG------------------
ddiscoideum           59 NTFANYWDEQLALFPVGQDSTFTCTTRVRLSKQEAN-------------C---------T
gmar_4_KAF0502320     59 DNLDTFWGANEIQYPSDSAGYAFFTTRASA------RNYPNPP-------C-NSLRT---P
consensus             61        vwd  dy  P        vmt    t  q    c e  c     c  g  tg c
```

Journal of Experimental Biology • Supplementary information

```
                     121 .......130.......140.......150.......160.......170.......180
mmus_1               119 VKTCEIFGWCP-VEVDLLHEAENFTLFIKNSISFPRFKVNRRNLNGTYMKKCLYHCPVFS
human_1              119 VKTCEIFGWCP-VEVDLLREAENFTLFIKNSISFPRFKVNRRNLNAAHMKTCLFHCPVFQ
mmus_4a              119 VKTCEVAAWCP-VENDFLKAAENFTLLVKNNIWYPKFNFSKRNITTSYLKSCIYNCPIFR
human_4.1            119 VKTCEVAAWCP-VEDDFLKAAENFTLLVKNNIWYPKFNFSKRNITTTYLKSCIYDCPIFR
ggal_4a              119 IKTCEVFAWCP-VEDDFLQGAENFTILVKNNIWYPKFNFSKRNISSSYLKNCIHDCPIFR
xlae_4               119 VKTCEIFAWCP-VENDFLNGAENFTVLIKNNIWYPKFQVSKRNISSSYLKTCQYDCPIFR
drer_4b              119 IKTCEVLSWCP-LENDLLSAAEDFTVLIKNNIQYPKFKFRKRNINSTYLKNCTFNCPVFR
hvul_4a              119 YRVCEIYGWCP-TETDLLDDAKEFTVLLKNEVIFPKFKVQRRNINSNYLKTCFHNCPIFK
nvec_4a              119 VKTCELYAWCP-IENDLLDAAKNFTLLIKNSVQFPKFSESRRNINDSHLKSCNYHCPVFR
amil_4a              119 KKVCEIYSWCP-VEIDLLGAAKDFTLLVKNNVQFPKFQETARNINTSYLKGCHYDCPIFK
tadh_x1              119 EMTCEIYSWCP-LEYDFLGEAVNFTVLIKNSIAFPKFNVRRSNINSEDLKHCMYDCPILK
mmus_5a              120 RGTCEIFAWCP-VETKLLKDAEGFTIFIKNFIRFPKFNFSKANVNKHFLKTCHFSCPIFR
human_5.a            120 RGTCEIFAWCP-LETSFLKEAEDFTIFIKNHIRFPKFNFSKSNVDRSFLKSCHFGCPIFR
drer_5               120 TGTCEINAWCP-VEHGMLAKAENFTVYVKNFIKFPKFGFFKSNVNSTYLKTCRYDCPIFL
mmus_6a              120 HRTCEIWSWCP-VESGLLAQAKNFTLFIKNTVTFSKFNFSRSNADNTYFKHCLYDCPVFR
human_6.1            120 HRTCEIWSWCP-VESGLLAQAQNFTLFIKNTVTFSKFNFSKSNADPTYFKHCRYECPVFR
xlae_6a              120 QSTCEIYGWCP-VENERLNEAENFTLFIKNVVHFSKFNFSRANTDETYFKNCRYNCPVFQ
ggal_6a              120 HSTCEIFGWCP-VENDLLAEAENFTLFIKNTVNFTKFNFSKGNTDPTYFKTCMYDCPVFR
acal_4.2             119 EKVCQIFGWCP-LENDVLENAKNFTVFIKNNIEFPRFGVSRRNINDTALKTCRWKCPIFV
cgig_4               119 VSRCEIHAWCP-VENGVLEESKKFTVFIKNNIEFPKYSVKRRNLSNDYLQSCRYDCPIFT
Em0004g77a           119 THTCDVYAWCP-TEYEPKINAENFTVLIKNTIQFPNLNFFKKNYD---LANCTNSCPIFK
Efra14662_1          119 TRTCEVFAWCP-TEYEPKINAGNFTVLIKNSIQFPNLDFFKKNYD---LANCTTSCPIFK
Efra14663_5          119 TNTCEVYAWCP-TEKEPKINARGFTVLIKNSVQFPNLNRLRKNYD---LSSCGIWCPIFV
Aqu003384136         118 NETCQIRGWCP-VEDEPIIDTKDFTVLVKNSISFPKLSYRVRNINKSYLSGCQFDCPIFS
mmus_3               119 RRTCEIQGWCP-TEVDIMMEAENFTIFIKNSIRFPLFNFEKGNLTDKDIKKCRFHCPILR
human_3              119 LRTCEIQGWCP-TEVDIMMEAENFTIFIKNSIRFPLFNFEKGNLTARDMKTCRFHCPILR
ggal_3               119 LHTCEIRGWCP-PEVDVMLEAENFTLFIKNSVRFPLFGFEKANLSAGELQRCRFHCPILR
drer_3b              119 HKTCEIKGWCP-AEIDPMMEVENFTIFIKNSIRFPRFNFTKGNFNSSYIKGCNFDCPIFK
mmus_2d              120 SKTCEVSAWCP-VEDGLGKMAPNFTILIKNSIHYPKFKFSKGNIKSDYLKHCTFDCPIFR
human_2.d            120 SKTCEVFGWCP-VEDGLGTMAPNFTILIKNSIHYPKFHFSKGNITDGYLKRCTFHCPIFK
ggal_2a              120 GRSCEVLAWCP-LHGGLAEMAAQFTILIKNHVREPRFGFSKANIESHYLKSCTFNCPIFR
mmus_7a              117 RKTCEVSAWCP-TEEELLRSAENFTVLIKNNIHFPGHNYTTRNING----SCTFHCSIFR
human_7              117 QKTCEVSAWCP-IEAVLLNSAENFTVLIKNNIDFPGHNYTTRNINI----TCTFHCPIFR
ggal_7               118 HKTCEIKAWCP-VQGEVLRSSEDFTVFIKNNIHFPTFNYTVQNIN----TSCKFNCPIFR
drer_7               118 KKTCEVTAWCP-IENKLLAAAENFTVMIKNNIRFPAFNYIRRNIKDTDFKGCIYHCPIFR
spur_4               111 TTVCEVKAWCP-VEVDILEEAENFTVLIKNSISFPKFGFVKRNIDPMYLRSCRYSCPIFR
sros_x               119 TKRCMVYGWGP-VELSQMPQVKNFTMFIKNTIFFPHFQRKFGNTSASYLKSCLWNCPVFR
hvul_E               100 ---TDYSAI---SHSSYPMYPEEALVKIEHSISTSSLSASHRMMDGHPLKFLNSNVDKLY
nvec_E1               98 ---QEFVNI---REKKFTVNPEEVMIKVEHSIVATRFAASQRSMQGKIIQSIPATADKLT
Aqu003385281          97 ---DEFEGI---NETQFTLGPEHVLVKVDHAVVASRFAASKRQMRGALIRKLSI-PDKIT
Slac9476              98 ---DEYILK---REQDFTQGPEYVVLKVDHAVVASDPAASQRKLDNKPISHLSPGPDKVS
Em0004g666a          100 ---DHYGTY---SEEQYTKGPEYVVLKIDHAITASDSRASHRKLNGNPVRQLNSDPDKLS
Avas14669             99 --TYNFETI---TEKQFTRNPESTILKIDHSILSTQFGGSHRTMNGKIIRQISD-PDKLK
Avas10395             99 --TYNFETI---TEKQFTRNPESTILKIDHSILSTQFGGSHRTMNGKIIRQISD-PDKLK
ddiscoideum           97 DPTCKFVDE-PGSAKNYIADIESFTILIDHTMYASSSQFNAVDLDGDEVQ---IDPDIMT
gmar_4_KAF0502320    103 SDPCVF---KP-SNNNYIGGIENYTMMIEHSIRGEATAIRNGLMDGKTVYTTKTNGDIFT
consensus            121    cei  wcp  e         ftl ikn i f  f     nl    m c   cpv
```

Journal of Experimental Biology • Supplementary information

```
                     181 .......190.......200.......210.......220.......230.......240
mmus_1               178 LGYVVRSGQ-DFRSLAEKGGVVGITIDWECDLDCKPIYQFHGLYGSPGFNFRFARHNGTN
human_1              178 LGYVVQSGQ-NFSTLAEKGGVVGITIDWHCDLDCRPIYEFHGLYESPGFNFRFARHNGTN
mmus_4a              178 LGQIVAAGH-SFQEMAVEGGIMGIQIKWDCNLDCLPRYSFRRLDTSPGYNFRFAKYAGNE
human_4.1            178 LGKIVEAGH-SFQDMAVEGGIMGIQVNWDCNLDCLPRYSFRRLDTSPGYNFRFAKYAGNE
ggal_4a              178 LGKIVEAGQ-NFQEMAVEGGVMGLQINWDCNLDCVPKYSFRRLDNSPGYNFRFAKYSGIE
xlae_4               178 LGNIVKAGE-SFSDMAVQGGVMGIQINWNCDLDCVPKYSFRRLDNSPGYNFRFAKYNGVE
drer_4b              178 LGDIVTSGE-DFSIMALKGGIIGIFIDWSCDLDCVPKYSFRRLDNAPGYNFRYAKYENTE
hvul_4a              178 IKDVIQCND-DFEQVAYLGAVYGILVNWDCNLDCTPTYSTRRLDDSPGFNFRFANYDNKQ
nvec_4a              178 LGTMAEAGV-NFEKLAYKGGVMAIVITWNCNFDCEPKYSFRRLDDAPGYNFRFPRYNETL
amil_4a              178 FDKMFEANI-TFEDIAFQGGVMAIIIKWNCNFDCEPKYQFTRLDDAPGYNFRFAKYDNVL
tadh_x1              178 LGTIVNAEQ-DFNKIA-------------------------------------------NNKL
mmus_5a              179 LGSIVRAGA-DFQDIALKGGVIGIHIEWDCDLDCNPHYYFNRLDNSSGYNFRFARYHGVE
human_5.a            179 LGSVIRAGS-DFQDIALEGGVIGINIEWNCDLDCHPHYSFSRLDNSSGYNFRFARYAGVE
drer_5               179 VGDVINTGY-TFQDLATRGGSIGIGIEWNCDLDCNPEYSFTRLDSTSGYNFRFARYAGQT
mmus_6a              179 IGDLVAAGG-DFEDLALLGGAVGISIHWDCNLDCCPQYSFQLQQK--GYNFRTANHSGVE
human_6.1            179 IGDLVAAGG-TFEDLALLGGSVGIRVHWDCDLDCWPHYSFQLQEK--SYNFRTATHPGVE
xlae_6a              179 IQEIITAGQ-SFEELSVMGGVVAARIEWKCDLDCLPQYSFRLQDT----NFRTATYERKE
ggal_6a              179 IRDMVEAGE-TFGALALMGGSISVRIEWDCDLDCQPRYSFILLDR---YNFRPPSSTSIG
acal_4.2             178 LDDIARAGV-TFENMMMEGGVMQIVIDWTCNLDCVPEYTFRRLDKSRGFNFRFADRTGLQ
cgig_4               178 LQSIVETGS-RYDELSGQGGVIQIRIKWNCNLDCLPEYSFRRLDSSKGYNFRYSRNNGTE
Em0004g77a           175 LADIVNEPK-NFSNLATKGAVVVLTITWNCDLDCNPRYSARRLDTSGGFNFRFAKYNGVQ
Efra14662_1          175 LADIVNAPK-NYDTLATKGAVVVMTITWNCDLDCNPTYSARRLDTTGGFNFRFAKYQGTQ
Efra14663_5          175 LGDIVAPPQ-NFTQLATEGAVIVLTITWNCDLDCDPQYDARRLDAAGGFNFRFAKYQGTQ
Aqu003384136         177 LKQIVDSDG-YYESLATKGAVVGLEISWDCNLDCVPKYSARRLDNSNGFNFRYPRYNGDD
mmus_3               178 VGDVVKAGQ-DFAKLARTGGVLGIKIGWVCDLDCIPKYSFTRLDGSPGYNFRFAKYNGSE
human_3              178 VGDVVKAGQ-DFAKLARTGGVLGIKIGWVCDLDCIPKYSFTRLDSSPGYNFRFAKYNGSE
ggal_3               178 LGDVARAGQ-DFASLAATGGVLGIKIGWVCDLDCLPRYSFTRLDSSPGYNFRHARYNGTE
drer_3b              178 VGDVIRAQQ-NFTTLATKGGVIGIKIAWVCDLDCKPAYSFTRLDASPGYNFRYAKYNGTE
mmus_2d              179 LGFIVEAGE-NFTELAHKGGVIGVIINWNCDLDCNPKYSFRRLDPSSGYNFRFAKYGTTT
human_2.d            179 LGFIVEAGE-SFTELAHKGGVIGVIINWDCDLDCNPKYSFRRLDPSSGYNFRFAKYNGTT
ggal_2a              179 LGFLAEAGE-DFAVLAEKGGVIGVIISWDCNLDCNPRYSFRRLDPSPGYNYRFAKYNGTC
mmus_7a              172 LGDIFQAGE-NFTEVAVQGGIMGIEIYWDCNLDCRPRYSFRRLDDVPGYNFRYAKYNNVE
human_7              172 LGDIFRTGD-NFSDVAIQGGIMGIEIYWDCNLDCRPKYSFRRLDDYPGYNFRYAKYNNVE
ggal_7               173 LGDILQAKE-NFSEMAVKGGIIAIEIKWDCDLDCSPEYSFRRLDDYPGFSIRFARHDGTE
drer_7               177 LGDIVAAKE-KFSEMAVEGGVIGIQINWDCDLNCLPKYSNRRLDEYPGLNFRFARYNGVE
spur_4               170 LGTIVATGQ-SFRQMAIKGGVITIDIQWNCNLDCLPKYRFIRADEAGGFNFGFGKFNDTE
sros_x               178 IEDILSAGVKSFEDVMMAGAVITVQIRYDCNLDCAPEYRFTRIDESNGFNFRFARYGEVP
hvul_E               154 LKDFLKANI-SLDTYRQNGLVLRVTIDYSNAK-GKTEYTYS----NLYSDYRIKQENGTI
nvec_E1              152 VQELLNAGIDSLESFRRHGMVLHVGICYHNTETGNIEYSYH----IPYADYRINQVTRPE
Aqu003385281         150 LQELLEGGVSGLDSIRQRGVVIIVSIYYQNWFNSDIEYSYQ----IPYMDYNSKQLTGRK
Slac9476             152 LQQLLTAGVESLDSFRERGCVLRVTIFYENWYCSDIQYTYH----IPYMDYSVREVEQGQ
Em0004g666a          154 LIELLKAGVESLESFRKRGCVLQVNIFYHNWLSSNAQYEYR----VPYIDYSTKEIINPL
Avas14669            153 VEELLMAGINTLDSYRMRGLILKVSIYYTNHEHGQIRYTYR----VPLVDYQTKQLFHTN
Avas10395            153 VEELLMAGINTLDSYRMRGLILKVSIYYTNHEHGQIRYTYR----VPLVDYQTKQLFHTN
ddiscoideum          153 IGQLLSGGV-SLDSIRYDGVVLFVFITYSNTYTSDFKYVYS----IANTIYDVPETESIH
gmar_4_KAF0502320    159 VQDLLTSGA-NLDTYRSSGIVIAIVINYQN---DDIVYSYL----IDGNEYKVTENNSTD
consensus            181 l  vv  g   f  l   ggvv i i w c ldc p ysf         nfr
```

Journal of Experimental Biology • Supplementary information

```
                     241 .......250.......260.......270.......280.......290.......300
mmus_1               237 -RRHLKVFGIRFDILVDGKAGKFDIIPTMTTIGSGIGIFGVATVLCDLLLLPKRHYYKQK
human_1              237 -YRHLKVFGIRFDILVDGKAGKFDIIPTMTTIGSGIGIFGVATVLCDLLLLPKRHYYKQK
mmus_4a              237 -QRTLKAYGIRFDIIVFGKAGKFDIIPTMINVGSGLALLGVATVLCDVIVMKKRYYYRDK
human_4.1            237 -QRTLKAYGIRFDIIVFGKAGKFDIIPTMINIGSGLALLGMATVLCDIIVMKKRLYYREK
ggal_4a              237 -TRTLKAYGIRLDIIVFGKAGKFDVIPTMINIGSGLALFGVATVLCDIVVMKKRYFYREK
xlae_4               237 -SRTLKVYGIRFDILVFGTAGKFDIIPTMINIGSGAALFGVATVLCDMIVFKKRHYYREK
drer_4b              237 -TRTLKAFGIRFDVIVFGMAGKFNIVPTIVNVGAALALLNLMKVICDWFMLSDSEYYSKH
hvul_4a              237 -YRTLKAYGLKFEIIVTGKAGKFSVIPLFTNLGAGLALLGIAVVWCDFIVLKKRFIYKEY
nvec_4a              237 -YRTIKAYGIRFVVTVYGKGGKFSVIPLFLNIGSGLALLGIATVLCDVFVLRKKYFYREK
amil_4a              237 -YRTLKAYGIRFAILVYGEGGKFSPVPLFLNLGSGLALIGVATVLCDIVVLQKKYFYREK
tadh_x1              197 -HRDLKAYGIRFVFIIVGRAGRFSVVPLLLNIGSGLALLAIASVISDVVILKRRQYYRSK
mmus_5a              238 -FRDLKAYGIRFDVIVNGKAGKFSIIPTVINIGSGLALMGAGAFFCDLVLIRKSEFYRDK
human_5.a            238 -FRTLKAYGIRFDVMVNGKGA----------------------FFCDLVLIKKREFYRDK
drer_5               238 -YRNLKVYGIRFDILVNGKAGRFSIIPTVINIGSGLALMGAGVFACDMILMSKSSFYRET
mmus_6a              236 -TRSLKLYGIRFDILVTGQAGKFALIPTAITVGTGAAWLGMVTFICDLLLDREAGFYWRT
human_6.1            236 -ARTLKLYGIRFDILVTGQAGKFGLIPTAVTLGTGAAWLGVVTFFCDLLLDREAHFYWRT
xlae_6a              234 -YRDLKLYGFRFDISVTGEARKFGVVPTAVSLGTGCAFLGAATFLCDLILDKKASFYRSC
ggal_6a              235 -RRSLKQQQLRH----RGLARTWGMLS--------------NAHWSDLQEQQKSLVHRE-
acal_4.2             237 LYRNLKAYGVRFLVTVQGKAGKFSIVPLLLNIGSGMALLGVATIICDIMVIKAKNFYRDK
cgig_4               237 -FRTLKAYGIKFVISVSGQARKFAPVPFFTNVGSGLALLSIATIICDIVVLKARALYKEK
Em0004g77a           234 -YRTLKAYGISFVVELVGQGGKFSFTALIFKLGSTIALLGIAAVLSDMVVVQKRHLYYQE
Efra14662_1          234 -YRTLKAYGILFIVELAGQGGKFSLAALFLKLGSTVALLGIAAVLSDMVVVQKRHLYWEQ
Efra14663_5          234 -YRTLKAYGILFIVELAGQGGKFSLAALFLKLGSTVALLGIAAVLSDMVVVQKRHLYWEQ
Aqu003384136         236 -VRDVKVYGIKFEIVVTGEGRKFSFTTLVLALGSTIALLAIATTVTDVIALKKGTYYREK
mmus_3               237 -YRTLKAFGIRFDVLVYGNAGKFNIIPTIISSVAAFTSVGVGTVLCDIILLKGADHYKAR
human_3              237 -YRTLKAFGIRFDVLVYGNAGKFNIIPTIISSVAAFTSVGVGTVLCDIILLKGADQYKAK
ggal_3               237 -LRTLKAFGIRFDVLVYGNAGKFGIVPTLINTVAAFTSIGVGTVLCDIILLKGAEHYKAR
drer_3b              237 -YRTLKAYAIRFDVLVNGDAGKFDMIPTLINMVAAFTSVGVGTVLCDIILLKGADQYKAK
mmus_2d              238 -TRTLKAYGIRIDVIVHGQAGKFSLIPTIINLATALTSIGVGSFLCDWILMNKNKLYSHK
human_2.d            238 -TRTLKAYGIRIDVIVHGQAGKFSLIPTIINLATALTSVGVGSFLCDWILMNKNKVYSHK
ggal_2a              238 -TRVLKAYGIRVDVIVQGQAGKFSLIPTVITLATALTSVGLGSFLCDWVLMDKERRYSSR
mmus_7a              231 -KRTLKAFGIRFDILVFGTGGKFDIIQLVVYIGSTLSYFGLATVCIDLLICTVNEYYRK
human_7              231 -KRTLKVFGIRFDILVFGTGGKFDIIQLVVYIGSTLSYFGLAAVFIDFLICVVNEYYRK
ggal_7               232 -QRTLKAYGIRFDVLVFGMGGQFKLIELFTFIGSTIAYFGLAVTIIEMCFIQVCENVIRK
drer_7               236 -QRTLKMYGIRFDVMVFGKAGKFSIIQLIIYIGSTLSYYAITTIFLDWLIKEAKQNYTER
spur_4               229 -YRDLKAYGILFQVKITGVAGKFDIVPLMLNFASGVALLSLATVMCDVVVLKKRKYYKEA
sros_x               238 -SRDLKVYGLRFVFIVSGTAGKFDFVPLLVTFGSGLGLLSLATIVADLLVLRNAEFYYDR
hvul_E               208 CKRVVKRYAVRIEFVQVGKIGEYSMSNLILQIVSLMGLLTLATTIIDVAAVPDKGIYRQY
nvec_E1              208 -KRLFKRYGIKIEFHQSGKLGRFSLPALLLKLVSGVGLLTLTATIVDTAALPDRFQYREF
Aqu003385281         206 -WQLLKRYSVRVEFQQTGSLGMFSFSSLLLKLVSGVGLLTLTATIIDTVALPNRMLYRKH
Slac9476             208 -QRLFKRYAVRLEFIQAGSLGMFSLTTLLLKLVSGVGLL---------------------
Em0004g666a          210 -ERLLKRYALRIEIVQSGSLGIFSLSALLSTIVNGNIVFTIAIWIIEKIAYQS---YIEE
Avas14669            209 -KRLLKRYSLRMIFEQEGSLGKTSLNALVYQLIAGTSLLTLLATVIDVVAIPT---YSRY
Avas10395            209 -KRLLKRYSLRMIFEQEGSLGKTSLNALVYQLIAGTSLLTLLATVIDVVAIPT---YSRY
ddiscoideum          208 -SRLLKRHGIRVIFIQTGTIGSFHFQTLLLTLVSGLGLLAVATTVVDQLALPQRKSYSSL
gmar_4_KAF0502320    211 GSITLDRHGIRFVFQQNGSIGVFDFVSLLTSLVASIALLKVAELIVEIIMLPEKDVYEDV
consensus            241   r lk ygir  i v G agkf i      i      l va  l d lll      y
```

Journal of Experimental Biology • Supplementary information

```
                          301 .....
mmus_1                    296 KFKYA
human_1                   296 KFKYA
mmus_4a                   296 KYKYV
human_4.1                 296 KYKYV
ggal_4a                   296 KYKYV
xlae_4                    296 KYKYV
drer_4b                   296 KFKHL
hvul_4a                   296 KYQKI
nvec_4a                   296 KYLNV
amil_4a                   296 KYQYV
tadh_x1                   256 KYQTV
mmus_5a                   297 KFEKV
human_5.a                 275 KYEEV
drer_5                    297 KFEAI
mmus_6a                   295 KYEEA
human_6.1                 295 KYEEA
xlae_6a                   293 KYEEV
ggal_6a                   275 -YMES
acal_4.2                  297 KYLDV
cgig_4                    296 KYLQV
Em0004g77a                293 KYQVV
Efra14662_1               293 KYLMV
Efra14663_5               293 KYLMV
Aqu003384136              295 KYQKV
mmus_3                    296 KFEEV
human_3                   296 KFEEV
ggal_3                    296 KFEEV
drer_3b                   296 KFEEV
mmus_2d                   297 KFDKV
human_2.d                 297 KFDKV
ggal_2a                   297 KFEQV
mmus_7a                   290 KCESI
human_7                   290 KCESI
ggal_7                    291 KYETV
drer_7                    295 KFEAV
spur_4                    288 KFQNV
sros_x                    297 KYEIV
hvul_E                    268 VFDSS
nvec_E1                   267 VYEES
Aqu003385281              265 VYDES
Slac9476                      -----
Em0004g666a               266 EYDEA
Avas14669                 265 MMDSS
Avas10395                 265 MMDSS
ddiscoideum               267 KFQVT
gmar_4_KAF0502320         271 KFDET
consensus                 301 kf
```

Fig. S2. Shown on the following pages is a figure of the consensus sequence shading of the alignment of all P2X receptor amino acid sequences in the bioinformatic analysis at 0.75 sequence agreement (range 0-1), constructed in BoxShade. Darker shading indicates a greater degree of amino acid conservation among the sequences.

Table S1. Differential transcript expression of sponge (*E. muelleri*) P2X receptors at different developmental stages. Values shaded from least relative expression (green) to highest relative expression (red) using MS Excel 2016 Conditional Formatting. Genes used in bioinformatic analysis are outlined in bold.

gene_name	Em0003g54a	Em0004g666a	Em0004g75a	Em0004g76a	Em0004g77a
Description	P2X recept	P2X recepto	P2X purino	P2X purino	P2X purinoceptor 4
FPKM.Sp2St1	0.00	0.00	0.25	0.87	3.77
FPKM.Sp3St1_	0.00	0.00	0.19	0.86	4.47
FPKM.Sp5St1a	0.00	0.09	2.06	0.18	3.21
FPKM.Sp2St2	0.00	1.30	1.21	0.48	2.35
FPKM.Sp3St2	0.00	1.33	1.18	0.35	3.30
FPKM.Sp5St2	0.00	1.22	6.30	0.13	4.72
FPKM.Sp2St3	0.00	2.63	1.03	0.31	1.93
FPKM.Sp3St3	0.00	1.77	1.26	0.82	2.27
FPKM.Sp5St3	2.54	2.77	1.34	0.65	3.55
FPKM.Sp2St5	0.97	2.30	0.88	1.35	7.11
FPKM.Sp3St5	0.00	3.15	1.35	1.16	7.37
FPKM.Sp5St5	0.02	2.28	0.75	0.90	5.30

Table S2. Supplementary Excel file with species names, abbreviations, and NCBI reference numbers used in bioinformatic analysis.

Available for download at
https://journals.biologists.com/jeb/article-lookup/doi/10.1242/jeb.248010#supplementary-data

Dataset 1. Supplemental Excel file with experimental data. Excel sheet tabs are titled according to the experiments.

Available for download at
https://journals.biologists.com/jeb/article-lookup/doi/10.1242/jeb.248010#supplementary-data

Journal of Experimental Biology • Supplementary information

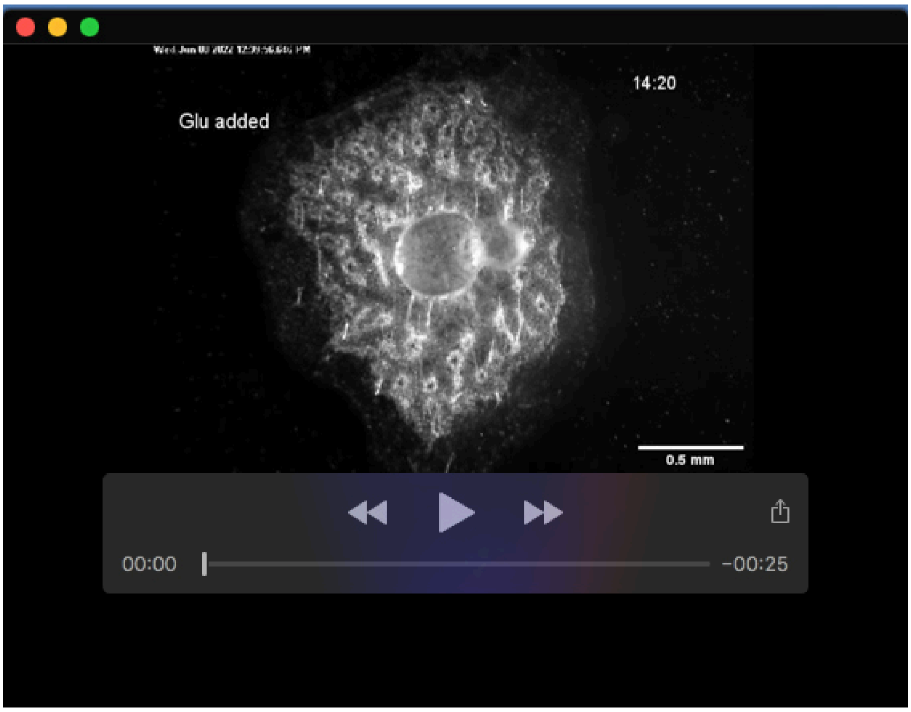

Movie 1. Sneeze in response to chemical stimulation (70 µM L-glutamate).

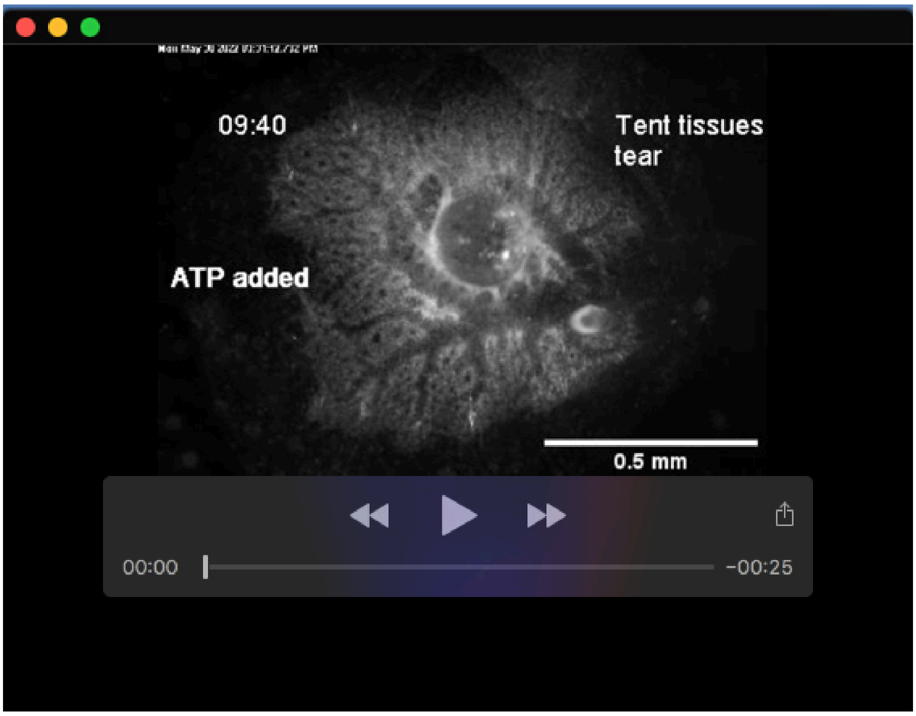

Movie 2. 200 µM ATP triggers a rapid and intense expansion of the excurrent canals (ECs) and prevents their constriction. The outer "tent" tissue is torn, along with parts of the ECs.

Journal of Experimental Biology • Supplementary information

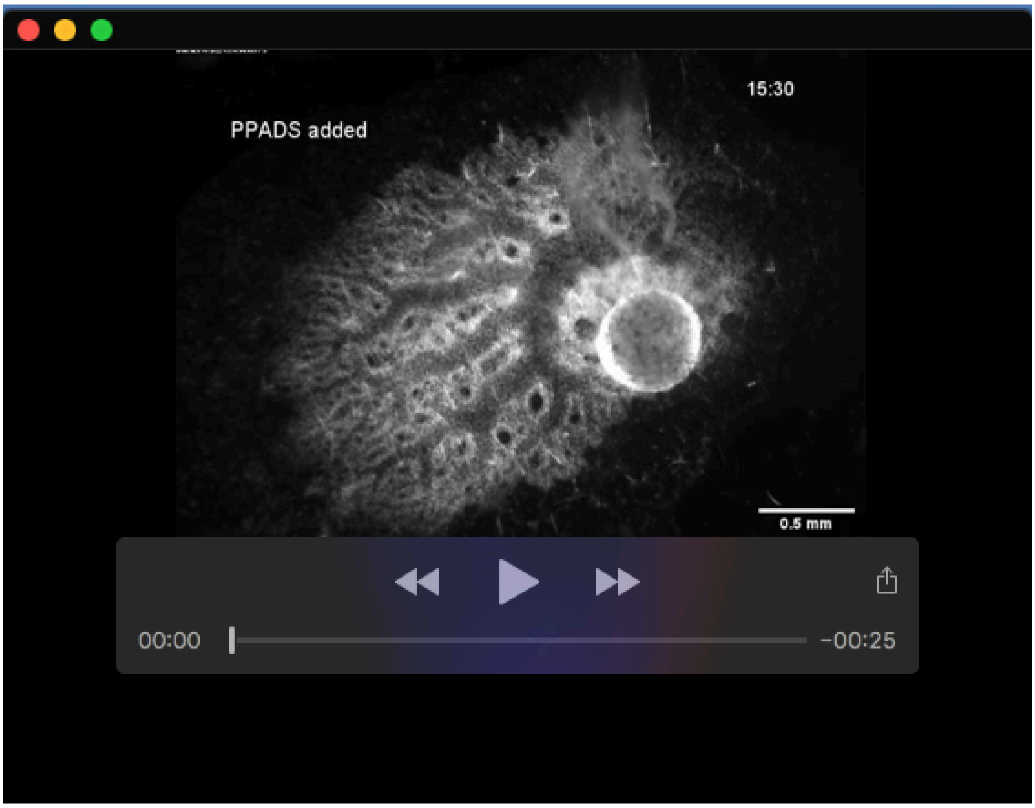

Movie 3. Pre-incubation with 100 µM PPADS for 20 min and application of 100 µM ATP. Sponges do not respond to ATP stimulation.

Additional movies are available at the Education and Research Archive of the University of Alberta (ERA): https://doi.org/10.7939/r3-n1k2-sm97

Journal of Experimental Biology • Supplementary information
